# Computational Repurposing of Janus Kinase Inhibitors as Potential Therapeutic Candidates for Alzheimer’s Disease

**DOI:** 10.3390/medicina62091674

**Published:** 2026-08-31

**Authors:** Ly Thi Huong Nguyen, Mai Thi Nguyen, Thai Uy Nguyen

**Affiliations:** 1 Institute of Theoretical and Applied Research, Duy Tan University, Hanoi 100000, Vietnam; 2 College of Medicine and Pharmacy, Duy Tan University, Da Nang 550000, Vietnam; 3 Faculty of Chemical Engineering, Ho Chi Minh City University of Technology (HCMUT), 268 Ly Thuong Kiet Street, Dien Hong Ward, Ho Chi Minh City 72511, Vietnam; nguyenmainhp@gmail.com; 4 Vietnam National University Ho Chi Minh City, Linh Trung Ward, Ho Chi Minh City 71308, Vietnam; 5 College of Health Sciences, VinUniversity, Hanoi 100000, Vietnam; uy.nt@vinuni.edu.vn; 6 Vinmec International Hospital, Hanoi 100000, Vietnam

**Keywords:** Alzheimer’s disease, JAK inhibitors, drug repurposing, molecular docking, network pharmacology, transcriptomic analysis

## Abstract

*Background and Objectives*: Alzheimer’s disease (AD) is the most common neurodegenerative disorder, and current therapies provide only limited symptomatic relief without effectively slowing its progression. Increasing evidence suggests that aberrant activation of the Janus kinase/signal transducer and activator of transcription (JAK/STAT) signaling cascade contributes to AD-associated neuroinflammation. This study investigated the therapeutic potential and molecular mechanisms of JAK inhibitors in AD using integrated bioinformatics and network pharmacology approaches. *Materials and Methods*: Potential anti-AD targets of JAK inhibitors were identified using the SwissTargetPrediction and GeneCards databases. Functional enrichment, protein–protein interaction (PPI) analysis, transcriptomic validation using public datasets, regulatory network construction, molecular docking, normal mode analysis (NMA), and absorption, distribution, metabolism, excretion, and toxicity (ADMET) prediction were performed to investigate the potential mechanisms of action of these drugs in AD. *Results*: Our analysis identified 163 shared targets between JAK inhibitors and AD. Enrichment analysis revealed that these genes were primarily involved in protein phosphorylation and were enriched in key signaling pathways, including the neurotrophin, phosphoinositide 3-kinase/protein kinase B (PI3K/Akt), and mitogen-activated protein kinase (MAPK) signaling pathways. PPI analysis identified AKT1, BCL2, SRC, STAT3, and TNF as five highly ranked hub targets across multiple topological algorithms. Transcriptomic validation confirmed significantly higher expression of these targets in the prefrontal cortex of individuals with AD compared with normal subjects. Molecular docking indicated that pacritinib and momelotinib showed relatively favorable predicted interactions with the hub proteins, while NMA revealed differences in the predicted flexibility of the docked complexes. Furthermore, ADMET prediction showed that pacritinib possesses favorable pharmacokinetic properties for the treatment of AD. *Conclusions*: Collectively, these findings provide mechanistic insights into the potential effects of JAK inhibitors in AD and identify pacritinib as a computationally prioritized candidate that warrants experimental validation in appropriate AD models. However, as this study is based solely on computational analyses without wet-lab validation, the findings should be considered hypothesis-generating in silico evidence, and the potential safety concerns of pacritinib require further investigation.

## 1. Introduction

Alzheimer’s disease (AD) is a progressive neurodegenerative condition marked by declining cognitive function, memory deficits, and neuropsychiatric symptoms [1]. It is the most common form of dementia, contributing to 60–70% of the total cases and resulting in an annual economic burden of trillions of dollars (World Health Organization, 2025). The key pathological characteristics of AD are the deposition of amyloid beta (Aβ) plaques and tau hyperphosphorylation, resulting in neurofibrillary tangles (NFTs) formation [2]. These abnormalities trigger neuroinflammatory responses, synaptic dysfunction, reduced neuronal plasticity, and progressive neurodegeneration, predominantly affecting the hippocampus and cerebral cortex [3]. Currently, commonly approved pharmacological treatments for AD consist of cholinesterase inhibitors (donepezil, rivastigmine, and galantamine) and the N-methyl-D-aspartate receptor antagonist memantine, which can only relieve symptoms and do not delay disease progression [4]. Moreover, long-term administration of these drugs may be accompanied by numerous side effects, such as nausea, diarrhea, and dizziness [5]. Several disease-modifying anti-amyloid therapies, including monoclonal antibodies lecanemab and donanemab, have been approved by the U.S. Food and Drug Administration (FDA) for the treatment of early stage AD. However, their clinical use remains limited by stage-specific indications, safety concerns such as infusion-related reactions and amyloid-related imaging abnormalities, including edema and effusions, and high treatment costs [6,7]. These limitations highlight the need to identify novel molecular targets and develop more effective and broadly applicable AD treatments.

Accumulating evidence suggests that chronic neuroinflammation plays a key role in the pathogenesis of AD [8]. The accumulation of Aβ plaques and NFTs activates microglia and astrocytes, the resident immune cells in the brain, leading to increased production of pro-inflammatory cytokines and chemokines, including IL-1β, IL-6, TNF-α, CCL2, and CXCL10, thereby triggering neuronal damage and accelerating disease progression [9]. The Janus kinase/signal transducer and activator of transcription (JAK/STAT) pathway is a major signaling pathway that regulates cytokine-driven inflammatory responses in neurodegenerative diseases [10,11]. The JAK family consists of four members: JAK1, JAK2, JAK3, and TYK2 (tyrosine kinase 2), which are activated by the binding of cytokines to receptors. JAK protein kinases phosphorylate STAT transcription factors to regulate the expression of genes related to neuroinflammation in various neurological disorders, including AD [12,13]. Analysis of post-mortem brain samples from 129 patients with AD and 101 healthy participants indicated that more than 50 genes in the JAK/STAT signaling pathway were dysregulated in AD compared to normal controls [14,15]. Furthermore, results from in vitro and in vivo AD models have demonstrated activation of the JAK/STAT pathway, with elevated levels of inflammatory cytokines and chemokines in blood and brain samples [14,16]. Therefore, modulating this signaling pathway may be a promising therapeutic strategy for suppressing neuroinflammation in AD.

Drug repurposing has emerged as a promising strategy for therapeutic discovery, exploring novel applications of approved drugs with well-established safety profiles [17]. Several selective JAK inhibitors have been approved by the U.S. Food and Drug Administration (FDA) for the treatment of chronic inflammatory diseases and cancers, such as psoriasis, atopic dermatitis, rheumatoid arthritis, and myelofibrosis [18]. These inhibitors primarily bind to the ATP-binding catalytic site of JAK family kinases, blocking their enzymatic activity and suppressing the activation of downstream signaling pathways [19]. Given the common inflammatory mechanisms between these disorders and neurodegenerative diseases, such inhibitors may hold therapeutic potential for AD by targeting neuroinflammatory pathways. Therefore, systematically investigating the repurposing potential of JAK inhibitors for AD may provide a solid foundation for elucidating their therapeutic effects as disease-modifying agents.

In the present study, we used an integrative computational approach that combined network pharmacology, transcriptomic data validation, and molecular docking to investigate the therapeutic potential of JAK inhibitors against AD-associated molecular targets. We focused on 11 FDA-approved JAK inhibitors with systemic administration, as these agents are more relevant for potential repurposing in AD, whereas topical JAK inhibitors are primarily used for localized dermatological conditions and are less suitable for treating neurodegenerative diseases, such as AD. In addition, the pharmacokinetic and safety profiles of the most promising candidates were assessed through absorption, distribution, metabolism, excretion, and toxicity (ADMET) predictions to evaluate their potential suitability for AD treatment. This study aimed to elucidate the potential mechanisms underlying the therapeutic effects of JAK inhibitors in AD and provide a basis for their repurposing as novel therapeutic candidates for AD.

## 2. Materials and Methods

### 2.1. Drug–Target Network Construction

Information on the 11 JAK inhibitors was retrieved from the DrugBank database (https://go.drugbank.com/) [20] (Table 1). Potential targets of the selected JAK inhibitors were predicted using SwissTargetPrediction (https://www.swisstargetprediction.ch/, accessed on 13 October 2025), and AD-related genes were obtained from the GeneCards database (https://www.genecards.org/, version 5.25, accessed on 13 October 2025) [21,22]. To improve prediction reliability, targets with a probability score > 0.1 in SwissTargetPrediction and disease-associated genes with a relevance score > 15 in GeneCards were included in subsequent analyses. These thresholds were selected as inclusive initial screening criteria, consistent with previous network pharmacology studies [23,24,25,26], to obtain a sufficiently broad set of potential drug targets and disease-related genes for the downstream network analysis. Overlapping targets between JAK inhibitors and AD were identified using Venny 2.1 (https://bioinfogp.cnb.csic.es/tools/venny/, accessed on 15 October 2025), and the common genes were visualized as a Venn diagram. A drug–target interaction network was constructed using Cytoscape 3.10.4 [27]. Network topology analysis was performed using the built-in Analyze Network tool to identify the key compounds within the network. To assess the robustness of target prediction, a sensitivity analysis was additionally performed using a stricter SwissTargetPrediction probability threshold of >0.5. The retained targets were subjected to further network analyses, and the resulting changes were compared with those obtained using the threshold of >0.1.

### 2.2. Gene Ontology (GO) and Kyoto Encyclopedia of Genes and Genomes (KEGG) Pathway Enrichment Analysis

Functional enrichment analyses of GO terms and KEGG pathways were performed using the Database for Annotation, Visualization, and Integrated Discovery (DAVID) 2021 platform (https://davidbioinformatics.nih.gov/) [28] to investigate the biological functions and signaling pathways associated with JAK inhibitor targets in AD in humans (Homo sapiens). Visualization of the top 10 enriched GO terms across biological processes, cellular components, and molecular functions, as well as the top 30 significantly enriched KEGG pathways (*p* < 0.001), was performed using the SRPlot web tool (https://www.bioinformatics.com.cn/srplot, accessed on 18 October 2025) [29]. The neurotrophin signaling pathway was retrieved from the KEGG database (https://www.genome.jp/kegg/pathway.html, accessed on 18 October 2025).

### 2.3. Protein–Protein Interaction (PPI) Network Analysis

A PPI network of the shared targets between JAK inhibitors and AD was constructed using the STRING database (version 12.0, https://string-db.org/) for Homo sapiens, with a minimum interaction confidence score of 0.4 [30]. The resulting network was imported into Cytoscape 3.10.4, and the hub proteins were determined using the cytoHubba plugin (version 0.1) [31]. The topological properties of the top 10 hub proteins were further evaluated using the Analyze Network tool in Cytoscape. Several cytoHubba algorithms, including Betweenness, Bottleneck, Closeness, Clustering Coefficient, Maximum Neighborhood Component (MNC), Radiality, and Stress, were used to evaluate the centrality of the proteins in the PPI network and to determine the top five hub proteins.

### 2.4. Transcriptomic Validation Using Public Microarray Datasets

For transcriptomic validation, three microarray datasets (GSE33000, GSE44768, and GSE44771) were downloaded from the Gene Expression Omnibus (GEO) database to evaluate the expression of target genes in control and AD samples [32]. These three datasets were generated using the same Rosetta/Merck Human 44k 1.1 microarray platform (GPL4372), representing the prefrontal cortex, cerebellum, and visual cortex, respectively. Notably, GSE44768 and GSE44771 represent different brain regions obtained from the same donor cohort. The clinical characteristics of the study populations are summarized in Table 2. Differential gene expression between the normal control and AD groups was analyzed separately for each dataset using the GEO2R web tool. For each gene, microarray expression values were obtained from the value column of the submitter-supplied GEO sample records through GEO2R. The expression data were normalized using the Force normalization option (quantile normalization), and boxplots were generated to assess the distribution of normalized expression values across samples. The resulting normalized expression values were used for subsequent statistical analyses and visualization. Expression values for each target gene were retrieved using the corresponding probe identifiers to ensure consistent gene-level comparisons across datasets. The probe IDs for *JAK1*, *JAK2*, *JAK3*, *TYK2*, *AKT1*, *BCL2*, *SRC*, *STAT3*, and *TNF* were 10023823514, 10023827011, 10025909502, 10025912720, 10025905959, 10023820570, 10025911688, 10023843357, and 10025909848, respectively. Receiver operating characteristic (ROC) curve analysis was used to assess the discriminative ability of the selected genes between AD and control within the selected datasets by calculating the area under the curve (AUC) and *p*-values.

### 2.5. Regulatory Network Construction

To explore the regulatory mechanisms of hub genes, transcription factors (TFs) were identified using the hTFtarget database (https://guolab.wchscu.cn/hTFtarget/, accessed on 18 October 2025), which predicts TF–target gene interactions [33]. Potential microRNAs (miRNAs) involved in the post-transcriptional regulation of hub genes were identified using the TargetScan database (https://www.targetscan.org/) [34]. The resulting TF–gene and miRNA–gene interaction networks were constructed and visualized using Cytoscape 3.10.4.

### 2.6. Molecular Docking Analysis

Molecular docking was performed using AutoDock Vina v1.5.7 to evaluate the binding interactions between the five most promising JAK inhibitors and five hub proteins [35]. The three-dimensional (3D) structures of the ligands and human target proteins were retrieved from PubChem (https://pubchem.ncbi.nlm.nih.gov/) and the Research Collaboratory for Structural Bioinformatics Protein Data Bank (RCSB PDB) (https://www.rcsb.org/), respectively [36,37]. Docking simulations were conducted with the energy range set to 4 kcal/mol and the exhaustiveness parameter set to 32. To validate the docking protocol, each protein was re-docked with its co-crystallized native ligand, and the accuracy of the predicted binding pose was assessed by calculating the root mean square deviation (RMSD) of heavy atoms relative to the experimental crystal structure using the “align” command in PyMOL v3.1.3. The grid box parameters used for each target protein are listed in Table 3. For each protein–ligand pair, nine docking poses were generated, and the conformation with the lowest binding energy was selected for further analysis. Protein–ligand interactions were visualized in 3D and 2D formats using PyMOL and Discovery Studio Visualizer v25.1.0 [38,39].

### 2.7. Normal Mode Analysis (NMA)

NMA was performed using the iMODS server to assess the flexibility and structural stability of the protein–ligand complexes using a coarse-grained modeling approach [40]. The docked complexes with the lowest binding energies were uploaded to the iMODS server in PDB format, and their dynamic properties were assessed based on deformability, B-factors, and eigenvalues.

### 2.8. Absorption, Distribution, Metabolism, Excretion, and Toxicity (ADMET) Prediction

The pharmacokinetic and toxicity profiles of the JAK inhibitors were predicted using the pkCSM web server based on their SMILES structures (https://biosig.lab.uq.edu.au/pkcsm/, accessed on 20 October 2025) [41]. The analysis included key parameters related to absorption (Caco-2 permeability and intestinal absorption), distribution (blood–brain barrier (BBB) and central nervous system (CNS) permeability), metabolism (inhibition of cytochrome P450 (CYP) enzymes, including CYP1A2, CYP2C9, CYP2C19, CYP2D6, and CYP3A4), excretion (total clearance and renal organic cation transporter 2 (OCT2) substrate), and toxicity (Ames mutagenicity and hepatotoxicity).

### 2.9. Statistical Analysis

Statistical analyses were performed using GraphPad Prism v.10 (GraphPad Software, San Diego, CA, USA). Multiple two-tailed *t*-tests were performed to compare gene expression between the normal control and AD groups. Subsequently, we applied the Benjamini-Krieger-Yekutieli (BKY) two-stage linear step-up method to adjust for multiple comparisons, maintaining the False Discovery Rate (FDR) at a 5% threshold. Statistical significance was reported as FDR-adjusted *p*-values (q-values), with a q-value of <0.05 considered statistically significant.

## 3. Results

### 3.1. Expression of JAK Members in Different Brain Regions in Control and AD Subjects

Three microarray datasets, GSE33000, GSE44768, and GSE44771, were used to investigate the mRNA expression of *JAK1*, *JAK2*, *JAK3*, and *TYK2* in the prefrontal cortex, cerebellum, and visual cortex, respectively, in healthy individuals and in patients with AD. After performing quantile normalization with GEO2R, the samples exhibited similar expression value distributions across the three datasets, suggesting that the data distributions were generally consistent post-normalization (Figure S1). Figure S2 indicates that the expression levels of *JAK1* and *TYK2* were significantly higher in the AD group than in the control group in all three datasets (q < 0.0001). However, JAK2 expression was significantly altered only in the visual cortex of the AD group (q = 0.0022), whereas JAK3 expression in the cerebellum did not differ significantly between the two groups (q = 0.4861). These results imply that dysregulation of JAK family members, particularly *JAK1* and *TYK2*, at the transcriptional level, may be associated with AD-related molecular changes. However, because JAKs are protein kinases, mRNA expression alone does not necessarily reflect protein abundance or kinase activity, and the absence of transcriptional changes does not exclude their functional involvement. These findings provide a transcriptional rationale for further investigation of JAK signaling and the therapeutic potential of JAK inhibitors in AD.

### 3.2. Screening for Target Genes of JAK Inhibitors Against AD

**Figure 1:**
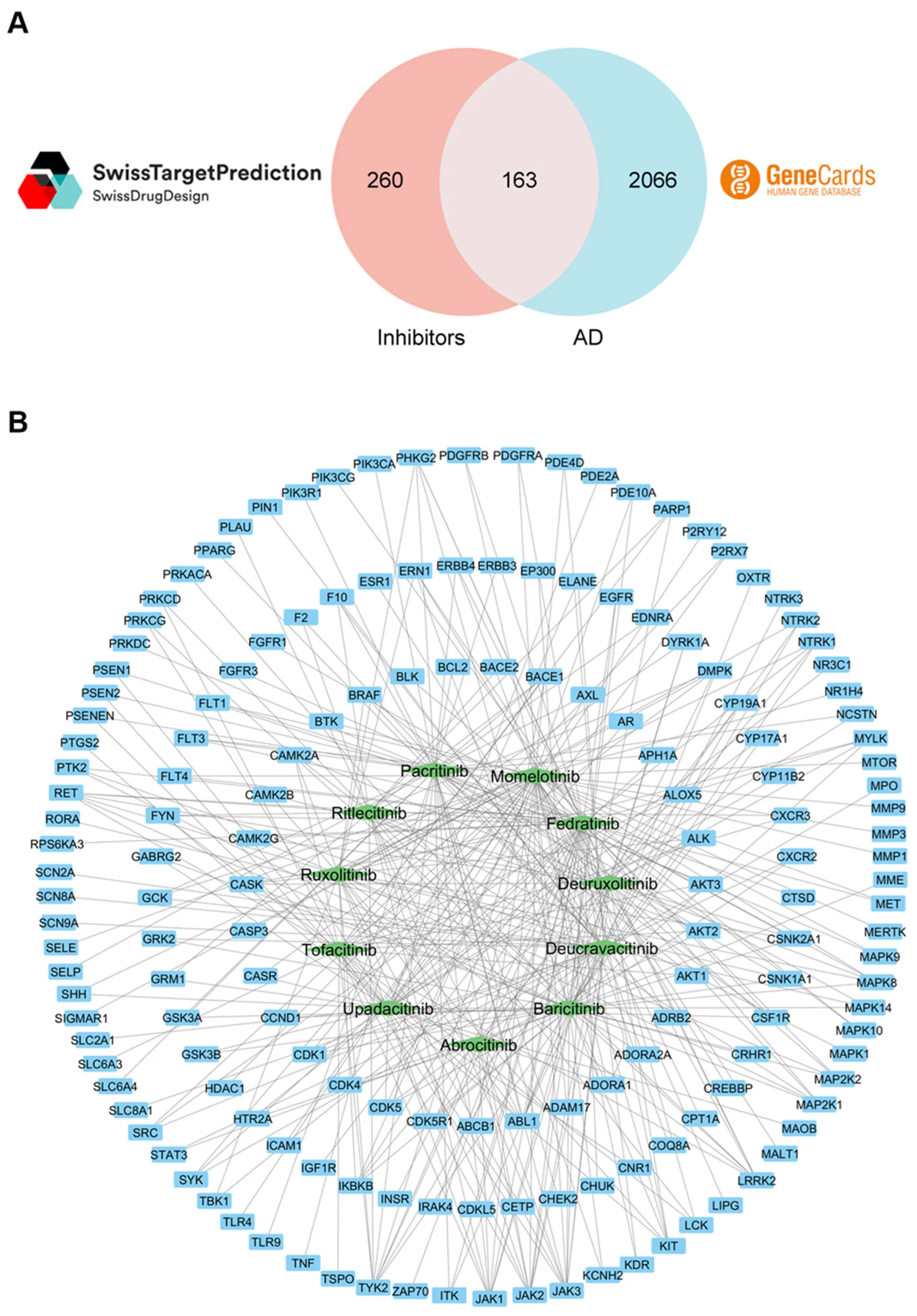
Construction of the drug–target network. (**A**) Venn diagram of overlapping targets between JAK inhibitors and AD. (**B**) Drug–target network constructed using Cytoscape, with green ellipses representing drugs and blue rectangles representing targets.

Eleven oral JAK inhibitors were selected to examine their potential effects on AD. These drugs have been approved by the FDA for treating cancer and inflammatory skin conditions. Using SwissTargetPrediction, we found that these 11 inhibitors targeted 426 genes (probability > 0.1) (Table S1). Data retrieval from the GeneCards database indicated 2219 AD-related genes with relevance scores ≥ 15 (Table S2). Our results revealed 163 overlapping targets between JAK inhibitors and AD (Figure 1A). Figure 1B shows the drug–target network comprising 174 nodes (11 drugs and 163 targets) and 357 edges. Table 4 presents the topological analysis of JAK inhibitors in the drug–target network. The results showed that baricitinib, momelotinib, fedratinib, pacritinib, and upadacitinib ranked highly in terms of degree, betweenness centrality, and closeness centrality, suggesting that these drugs may have greater potential relevance to AD than the other JAK inhibitors.

As a sensitivity analysis, a stricter SwissTargetPrediction probability threshold (>0.5) was additionally applied, reducing the number of predicted targets from 426 to 160, of which 58 overlapped with AD-related genes (Table S3), compared with 163 overlapping targets identified using the primary > 0.1 threshold. These results show that target selection varied considerably with the probability threshold applied. However, the primary threshold (>0.1) was retained for subsequent analyses to enable a broader investigation of potential drug–disease targets.

### 3.3. GO Enrichment and KEGG Pathway Analysis

**Figure 2:**
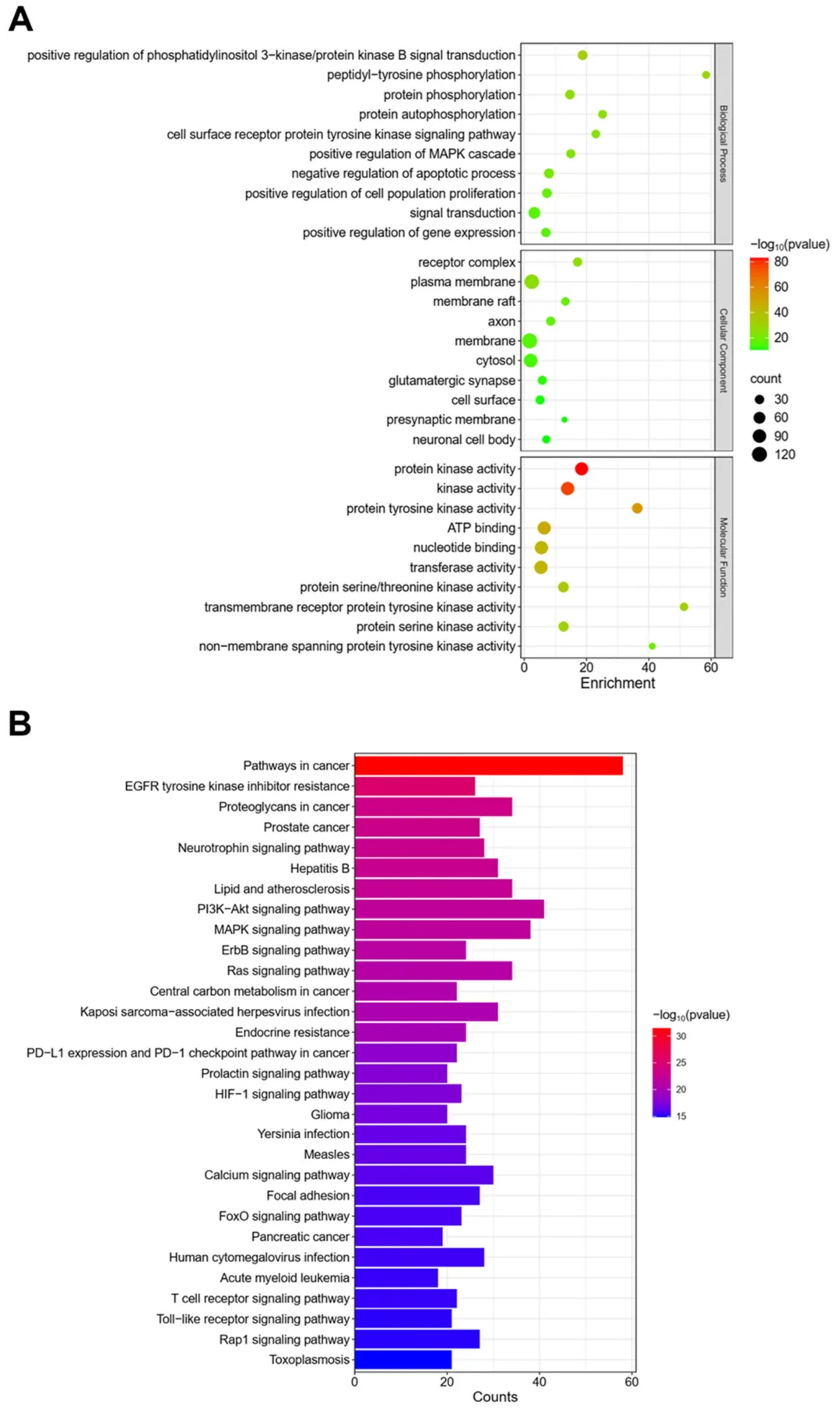
GO and KEGG pathway enrichment analyses. (**A**) Top 10 GO biological process, cellular component, and molecular function terms for AD-related target genes. (**B**) Top 30 KEGG pathways for AD-related target genes.

DAVID functional annotation was employed to analyze the GO terms and KEGG pathways associated with the 163 potential JAK inhibitor targets. The analysis revealed 782 biological process (BP), 114 cellular component (CC), and 141 molecular function (MF) terms related to these targets (Tables S4–S6). Figure 2A shows the top ten significant terms for each subcategory. The main BP terms included positive regulation of phosphatidylinositol 3-kinase/protein kinase B (PI3K/Akt) signal transduction, peptidyl-tyrosine phosphorylation, and protein phosphorylation. The target genes were involved in several CC terms, such as receptor complex, plasma membrane, and membrane raft. Protein kinase activity, kinase activity, and protein tyrosine kinase activity are key MF terms associated with potential targets.

KEGG pathways related to the target genes were also analyzed. A total of 176 KEGG pathways were identified (Table S7), and the top 30 pathways are shown in Figure 2B. Several KEGG pathways, including the neurotrophin, PI3K-Akt, and MAPK signaling pathways, may be closely associated with AD pathogenesis. Figure S3 and Table S6 indicate that the 28 target genes of JAK inhibitors encode 21 proteins involved in the neurotrophin signaling pathway, highlighting the significant role of this pathway in mediating the effects of JAK inhibitors in AD.

### 3.4. PPI Network Construction

**Figure 3:**
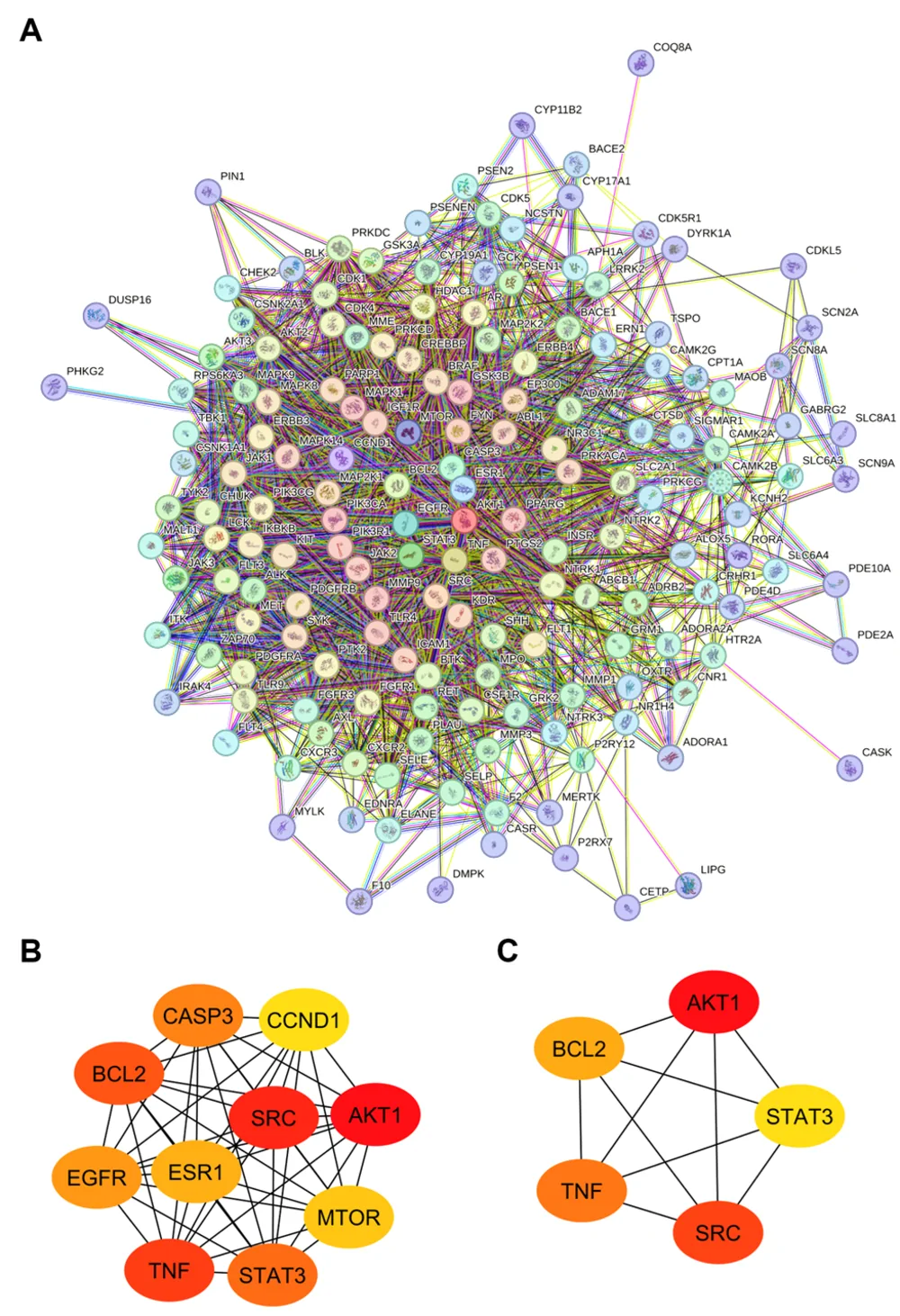
Construction of the PPI network of AD-related targets. (**A**) PPI network. (**B**) Subnetwork of the top 10 hub proteins. (**C**) Subnetwork of the top five hub proteins.

Protein interactions are essential for maintaining cellular homeostasis, and analyzing these interactions can help identify potential targets for the treatment of diseases. Figure 3A shows the PPI network of the potential targets associated with the 11 JAK inhibitors, consisting of 163 nodes and 2377 edges, with a PPI enrichment *p*-value < 10^−16^ and an average node degree of 29.2. Cytoscape with the cytoHubba plugin was used to identify and visualize hub proteins in the PPI network. The subnetworks comprising the top 10 hub proteins ranked by degree are shown in Figure 3B, and their topological parameters are presented in Table 5. Multiple cytoHubba algorithms, including Betweenness, Bottleneck, Closeness, Clustering Coefficient, MNC, Radiality, and Stress, were applied to further assess the centrality of the proteins. Table S8 shows that AKT1, BCL2, SRC, STAT3, and TNF consistently ranked among the top proteins across these algorithms and were therefore selected as the five key targets for further analyses (Figure 3C).

The stricter probability threshold (>0.5) used in the sensitivity analysis also altered the PPI network (Figure S4A), which consisted of 58 nodes and 384 edges, with an average node degree of 13.2. The top five hub proteins also differed, with only SRC retained from the primary analysis (Figure S4B), indicating that hub-target identification was sensitive to the selected prediction cutoff.

### 3.5. Transcriptomic Validation of the Hub Targets

**Figure 4:**
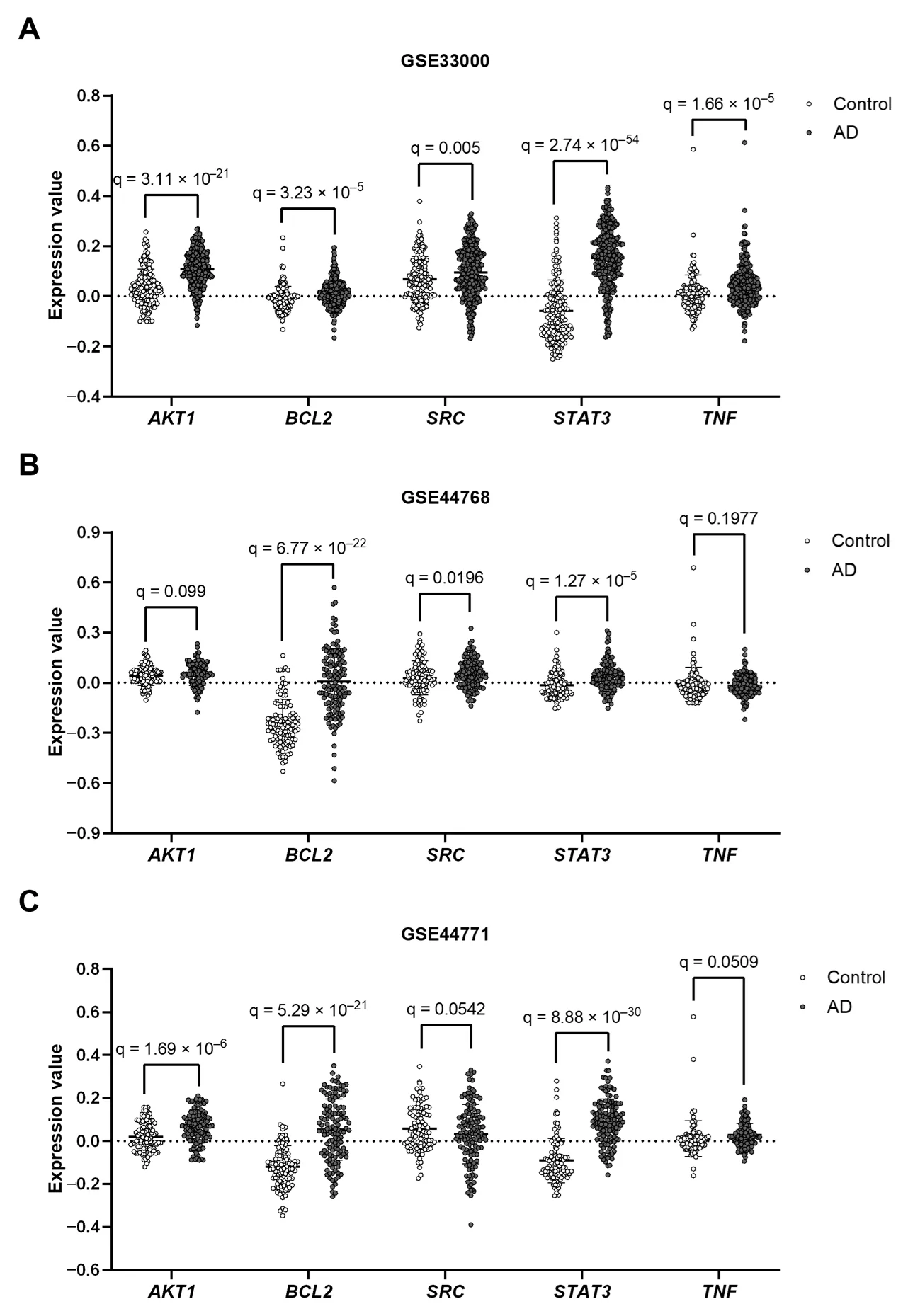
Transcriptomic validation of five hub targets in different brain regions in control and AD patients. The levels of *AKT1*, *BCL2*, *SRC*, *STAT3*, and *TNF* were retrieved and analyzed from the GEO datasets: (**A**) GSE33000 (prefrontal cortex), (**B**) GSE44768 (cerebellum), and (**C**) GSE44771 (visual cortex). Statistical analysis was conducted using multiple unpaired *t*-tests with Benjamini-Krieger-Yekutieli FDR correction (q-value).

**Figure 5:**
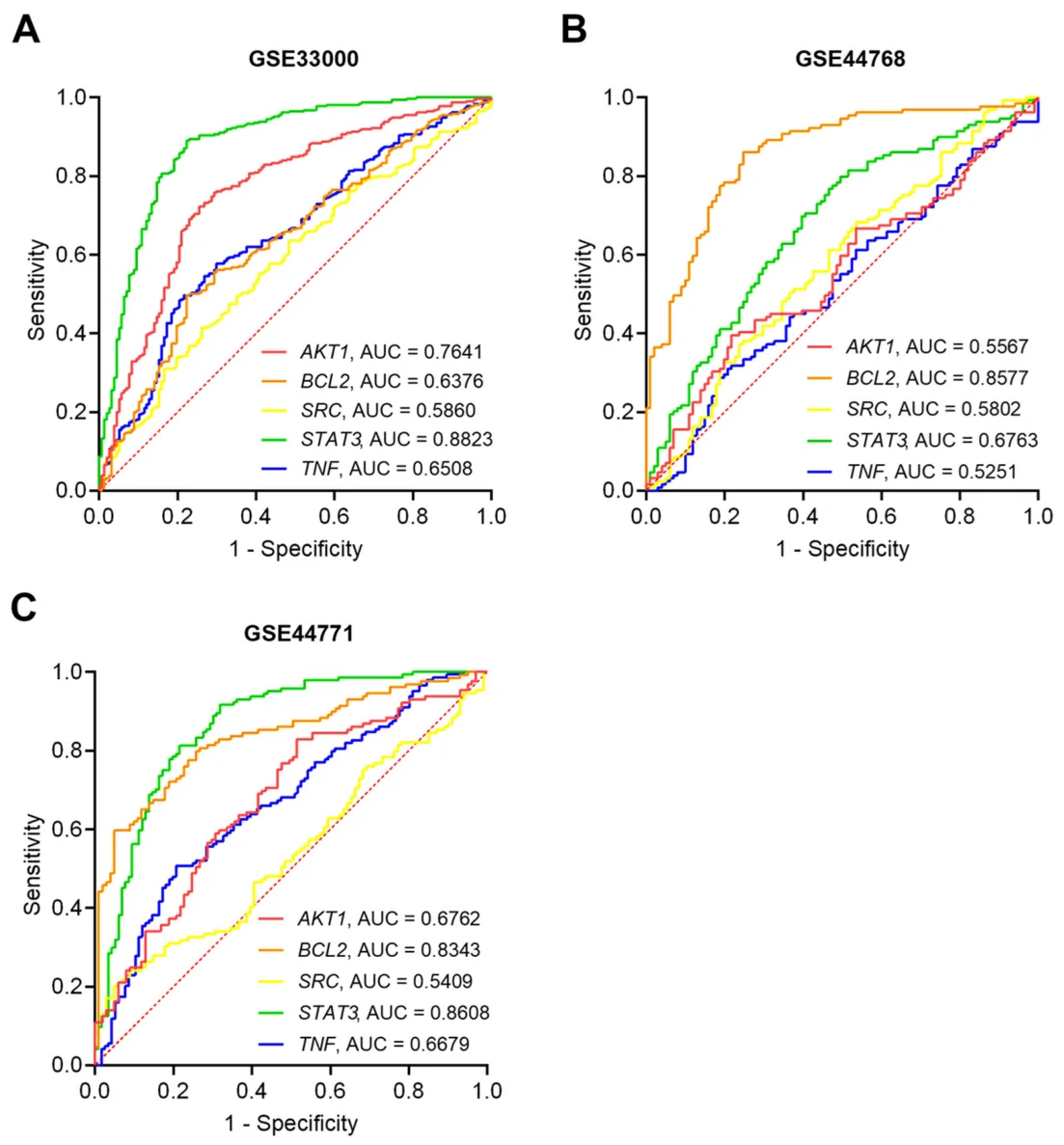
Discriminative ability of the five hub genes. Receiver operating characteristic (ROC) curve analysis was performed using GEO datasets: (**A**) GSE33000 (prefrontal cortex), (**B**) GSE44768 (cerebellum), and (**C**) GSE44771 (visual cortex).

To validate the five hub targets (*AKT1*, *BCL2*, *SRC*, *STAT3*, and *TNF*), microarray datasets from various brain regions of healthy controls and AD patients were analyzed. Figure 4A shows that the mRNA levels of all five targets were significantly increased in the prefrontal cortex (GSE33000 dataset) of patients with AD compared to those of healthy participants. However, as shown in Figure 4B, *AKT1* and *TNF* expression in the cerebellum (GSE44768 dataset) did not differ between the two groups, and a similar trend was observed for *SRC* and *TNF* expression in the visual cortex (GSE44771 dataset) (Figure 4C). These findings suggest that they may partially serve as therapeutic targets for JAK inhibitors in AD treatment, particularly in the prefrontal cortex. These region-specific differences in expression may reflect the heterogeneous progression of AD pathology across brain regions, with the prefrontal cortex being more susceptible to neuroinflammatory changes than other regions. ROC curve analysis was performed to evaluate the discriminatory ability of the hub targets between the AD and control groups within the selected datasets (Figure 5). The five target genes showed varying levels of discriminative ability, with AUC values ranging from 0.5251 to 0.8823. Notably, *STAT3* demonstrated the strongest discriminative ability across multiple transcriptomic datasets. These findings suggest the discriminatory ability of the identified genes between AD and control samples within the analyzed datasets, warranting further investigation of their biological significance and therapeutic relevance in AD.

### 3.6. Regulatory Network of the Hub Targets

**Figure 6:**
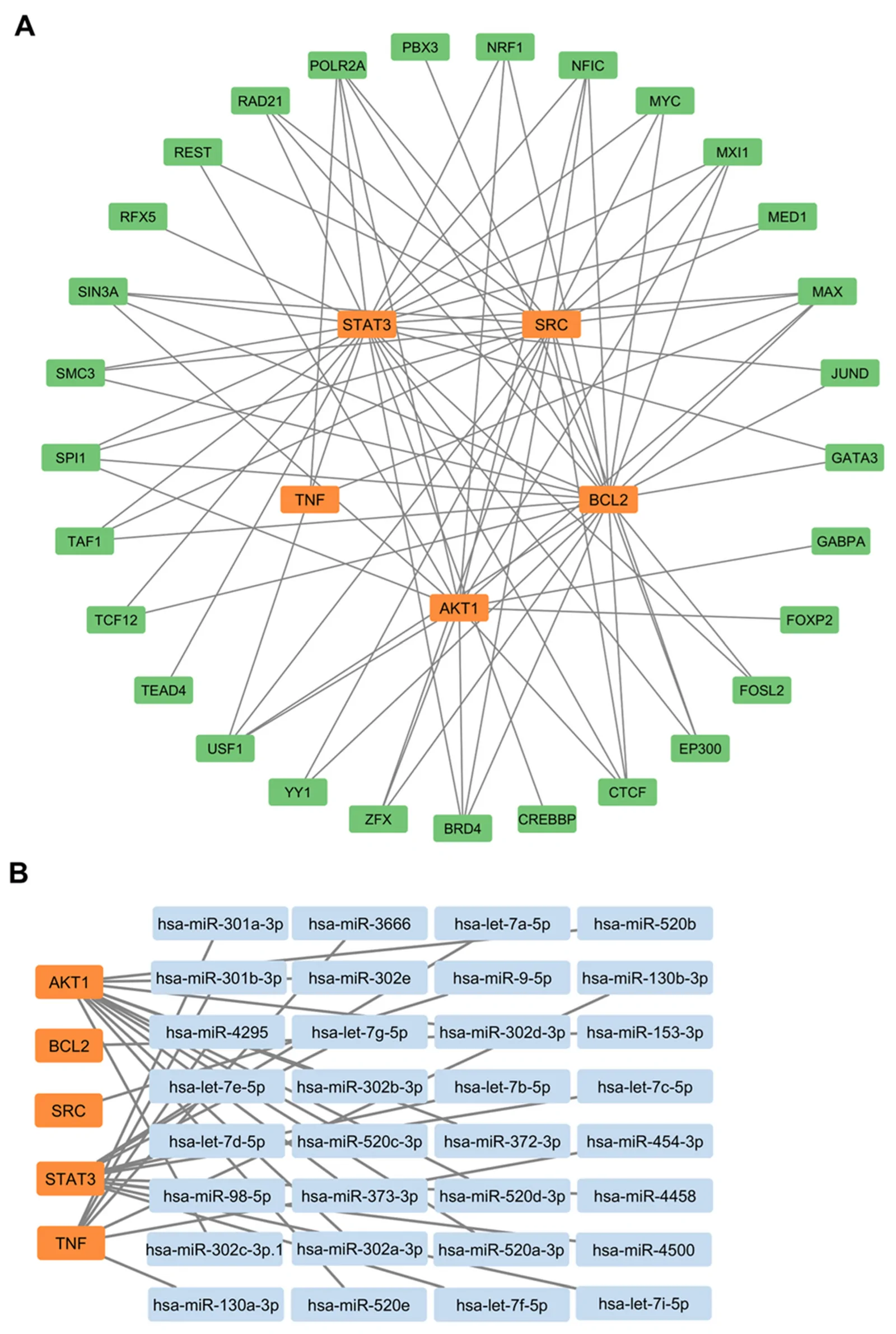
Construction of the regulatory network of the five hub genes. (**A**) mRNA–transcription factor network. (**B**) mRNA–miRNA network.

To better understand the upstream regulatory mechanisms of core targets, we examined their potential transcriptional and post-transcriptional regulators. Analysis using the hTFtarget database identified 29 transcription factors (TFs) that may regulate the five hub genes, indicating a complex transcriptional regulatory landscape (Figure 6A). Additionally, screening using the TargetScan database revealed 32 miRNAs that could target these core genes, highlighting an extensive post-transcriptional regulatory network (Figure 6B). Integrated regulatory networks, including mRNA–TF and mRNA–miRNA interactions, were constructed and visualized using the Cytoscape software. Overall, these findings suggest that hub targets are tightly regulated at multiple levels and that this layered regulation may play a key role in their expression and functional involvement in AD pathogenesis.

### 3.7. Molecular Docking Analysis of Potential Drug Candidates and Hub Proteins

Molecular docking analysis was conducted to investigate the potential interactions between the top five drug candidates (baricitinib, momelotinib, fedratinib, pacritinib, and upadacitinib) and the top five hub proteins (AKT1, BCL2, SRC, STAT3, and TNF). The docking protocol was validated by redocking five proteins with their native ligands. The results successfully replicated the experimental binding mode of native ligands with target proteins (Figure S5), achieving an RMSD below the 2.0 Å threshold (Table S9). This suggests that the docking parameters are suitable for further molecular docking studies with the specified drugs, demonstrating a high predictive capacity.

**Figure 7:**
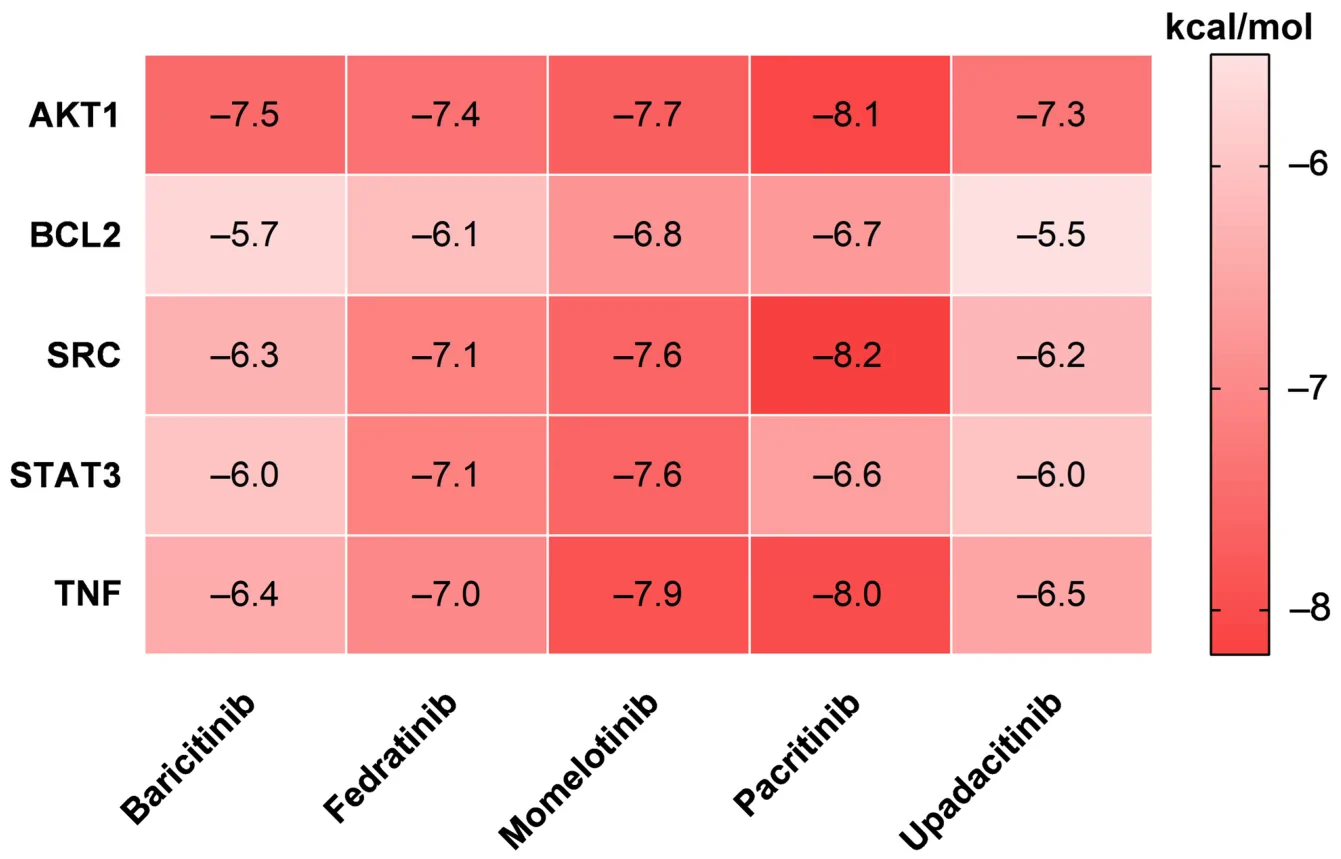
Docking scores between JAK inhibitors and the target proteins.

**Figure 8:**
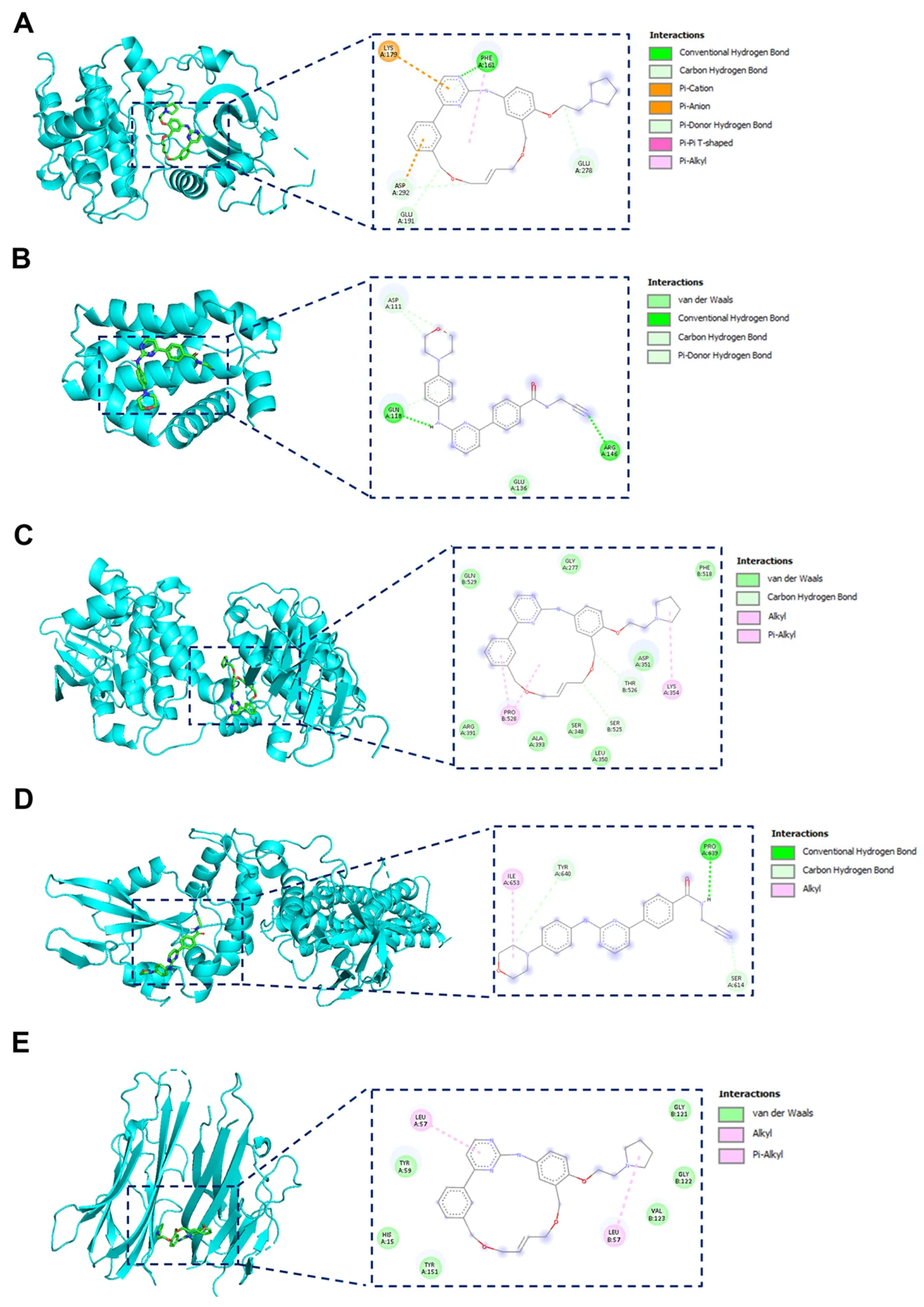
3D and 2D visualization of representative binding conformations of JAK inhibitors with their target proteins. (**A**) Pacritinib–AKT1. (**B**) Momelotinib–BCL2. (**C**) Pacritinib–SRC. (**D**) Momelotinib–STAT3. (**E**) Pacritinib–TNF.

The docking scores for the JAK inhibitors and target proteins were negative and almost all below −6.0 kcal/mol, indicating strong binding affinity (Figure 7). Pacritinib exhibited the lowest binding energies with AKT1, SRC, and TNF, whereas momelotinib showed the strongest affinity for BCL2 and STAT3, suggesting that they may be the most promising candidates for AD treatment by targeting these proteins. The 3D and 2D binding modes of the representative conformations with the lowest docking scores are shown in Figure 8. The typical interactions between ligands and proteins are van der Waals forces, conventional hydrogen bonds, carbon-hydrogen bonds, and hydrophobic interactions (Pi-alkyl and alkyl) at the active site residues of the proteins. Table 6 lists the interaction types, specific residues involved, and corresponding bond distances for the representative protein-compound complexes.

### 3.8. NMA of Potential Drug–Hub Protein Complexes

NMA of the ligand–protein complexes was performed to examine the stability and mobility of the docked complexes between pacritinib and the five proteins. Figure S6 shows the molecular mobility patterns for each complex, with arrows indicating the direction of motion; longer arrows indicate a greater motion. Deformability and B-factor profiles were analyzed to determine the mobility of the docked complexes. As shown in Figure S7, the five complexes between pacritinib and the target proteins exhibited limited structural flexibility, with several moderate peaks in the deformability plots and broadly comparable trends between the predicted NMA and PDB-derived B-factor profiles. The eigenvalue represents the energy required for the protein structure to move along a specific normal mode, with smaller eigenvalues indicating lower motion stiffness and larger eigenvalues denoting stiffer systems. Table S10 shows that the eigenvalues of the five complexes ranged from 2.11 × 10^−5^ to 5.01 × 10^−4^, indicating differences in the stiffness of the analyzed conformations. Taken together, these NMA results provide qualitative insights into the predicted flexibility of the docked complexes.

### 3.9. ADMET Prediction of Potential Drugs

The pharmacokinetic profiles of the five inhibitors were predicted using the pkCSM platform (Table 7). All the compounds exhibited favorable absorption properties. The predicted Caco-2 permeability (log Papp) values ranged from 0.349 to 1.356, and the predicted intestinal absorption rates were relatively high for all compounds (79.6–94.07%), suggesting acceptable intestinal permeability and efficient gastrointestinal absorption. Pacritinib exhibited the highest Caco-2 cell permeability (1.356) and intestinal absorption rate (94.07%), suggesting a favorable oral bioavailability. The predicted blood–brain barrier (BBB) permeability values (log BB) ranged from −1.346 to −0.267, and the predicted central nervous system (CNS) permeability values (log PS) ranged from −3.606 to −1.924. Among the five JAK inhibitors, pacritinib showed relatively better BBB penetration and CNS permeability, indicating its greater ability to reach the brain and its potential for treating neurological diseases such as AD.

Cytochrome P450 (CYP) interaction prediction indicated that the compounds exhibited distinct metabolic profiles. Momelotinib and upadacitinib were predicted to inhibit CYP1A2, and fedratinib and momelotinib were predicted to inhibit CYP2C19. Fedratinib and pacritinib exhibited inhibitory potential against CYP2C9, whereas fedratinib, momelotinib, and pacritinib were predicted to inhibit CYP3A4. None of the compounds were predicted to inhibit CYP2D6, indicating a low risk of metabolic interactions with this enzyme. The predicted total clearance values (log mL/min/kg) ranged from 0.344 to 0.875. Baricitinib showed the highest predicted clearance (0.875), whereas momelotinib showed the lowest predicted clearance (0.344). No compound was predicted to interact with the renal OCT2 transporter as a substrate, implying minimal involvement of this transporter in renal excretion.

Ames toxicity prediction indicated that momelotinib may be mutagenic, whereas the other compounds were predicted to be non-mutagenic. However, all five compounds were predicted to be hepatotoxic, suggesting that liver safety at adjusted doses should be carefully evaluated in future studies. Overall, the ADMET predictions indicated that these inhibitors have a favorable pharmacokinetic profile. Pacritinib exhibits good intestinal absorption and the ability to cross the BBB and enter the CNS, suggesting that it is a promising candidate for further investigation as a treatment for AD. However, potential risks, such as hepatotoxicity and CYP inhibition, should be carefully considered in future research.

## 4. Discussion

The repurposing or repositioning of clinically approved drugs has become a promising approach in drug discovery, offering faster and more cost-effective alternatives to traditional strategies. Several drugs have been successfully repurposed and approved by the FDA, including thalidomide (a sedative agent) for multiple myeloma and aspirin (an analgesic drug) for cardiovascular diseases [42,43], highlighting the reliability of this approach for therapeutic investigations. In this study, we systematically demonstrated the potential of repurposing FDA-approved JAK inhibitors for AD treatment by integrating network pharmacology, molecular docking, and transcriptomic validation.

Inhibition of the JAK/STAT signaling pathway improves Aβ-induced cognitive impairment in AD mice by suppressing inflammatory responses and apoptosis in the brain [44,45]. Network pharmacology analysis in the current study indicated that the 11 selected JAK inhibitors targeted multiple AD-related genes, suggesting their potential for AD treatment by modulating these targets. Topological analysis revealed that baricitinib, momelotinib, fedratinib, pacritinib, and upadacitinib were the top five candidates with the highest rankings in the drug–target network, suggesting that they may target more genes/proteins to exert their effects on AD than other drugs. Among these five inhibitors, only baricitinib has been extensively investigated and has exhibited beneficial effects on several neurological disorders, including AD, amyotrophic lateral sclerosis, and sepsis-induced cerebral and cognitive injury, by alleviating neuroinflammation and oxidative stress [46,47,48]. The effects of the remaining drugs on AD have not yet been reported, presenting a promising opportunity for further research to identify new therapeutic applications for these inhibitors in AD treatment.

GO enrichment analysis indicated that JAK inhibitors mainly regulate protein kinase activity and the phosphorylation process. JAK inhibitors directly interfere with the activities of the JAK family of non-receptor tyrosine kinases, including JAK1-3 and TYK2; thus, they may also interfere with the activities of other protein kinases. In addition, KEGG pathway analysis revealed that JAK inhibitor target genes and proteins are involved in the MAPK and PI3K/Akt signaling pathways, which are crucial for protein phosphorylation in the regulation of neuroinflammation, oxidative stress, and neuronal apoptosis. The MAPK pathway, particularly p38 MAPK signaling, has been increasingly implicated in AD pathogenesis. Its activation is associated with the accumulation of Aβ plaques and NFTs, as well as increased neuroinflammation, neuronal cell death, and synaptic dysfunction, thereby contributing to the development and progression of AD [49]. Neflamapimod, a selective p38α kinase inhibitor, has been reported to attenuate Aβ- and Tau-associated toxicity and neuroinflammation and improve synaptic dysfunction in patients with early stage AD, highlighting the potential of targeting p38 MAPK signaling as a therapeutic strategy for AD [50]. A previous study demonstrated that ruxolitinib, a Jak 1/2 inhibitor, could inhibit the extracellular signal-regulated kinase (ERK) and p38 MAPK pathways to alleviate lipopolysaccharide-induced neuroinflammation in BV2 microglial cells and a mouse model, suggesting that JAK inhibitors may suppress MAPK signaling to ameliorate AD neuropathology [51].

The PI3K/Akt pathway may play multifaceted and context-dependent roles in AD that may vary across different cell types in the brain [52]. In neurons, activation of the PI3K/Akt signaling pathway promotes cell survival via multiple mechanisms. Akt phosphorylates the transcription factor FOXO and modulates the activity of p53 and Bcl-2 family proteins, thereby suppressing apoptosis and promoting neuronal survival [53]. Moreover, PI3K/Akt activation has been reported to alleviate Aβ toxicity and Tau hyperphosphorylation by inhibiting glycogen synthase kinase-3 beta (GSK-3β) activity, protecting neurons from injury and cell death [54]. Conversely, in AD, increased expression and activation of Toll-like receptors (TLRs) in microglia may activate the PI3K/Akt pathway, contributing to the production of pro-inflammatory cytokines, including IL-1β, IL-6, and TNF-α, as well as reactive oxygen species (ROS) [55]. Elevated levels of these mediators promote chronic neuroinflammation, oxidative stress, and mitochondrial dysfunction, ultimately contributing to neuronal damage and cognitive impairment. These findings suggest that both activation and inhibition of PI3K/Akt signaling may have beneficial effects in AD, depending on the cellular context. A previous study showed that the JAK inhibitor tofacitinib increased the phosphorylation of Akt and its downstream target GSK-3β, which was accompanied by reduced levels of the inflammatory cytokines TNF-α and IL-6 in the brain and improved cognitive function in Aβ-induced AD mice [45]. However, that study did not investigate whether tofacitinib affects biomarkers related to neuronal survival. In contrast, baricitinib, another JAK inhibitor, reduced the levels of phosphorylated PI3K, Akt, and mTOR, which are associated with decreased levels of neuroinflammation and oxidative stress markers, including NF-κB p65, TNF-α, IL-6, and malondialdehyde. These changes were associated with improved memory impairment in ovariectomized/D-galactose-induced AD rats [46]. Together, these findings highlight the context-dependent effects of JAK inhibitors on PI3K/Akt signaling in AD and suggest that different JAK inhibitors may modulate this pathway via distinct mechanisms. Therefore, the specific effects and underlying mechanisms of other JAK inhibitors in AD warrant further investigation.

KEGG pathway enrichment analysis indicated that the neurotrophin signaling pathway may be a crucial pathway in which JAK inhibitor targets are involved. Neurotrophins, such as NGF and brain-derived neurotrophic factor (BDNF), play key roles in AD development by regulating neurogenesis, synaptic plasticity, and neuronal survival [56]. Our analysis revealed that several key proteins in the neurotrophin signaling pathway, including TrkA and TrkB, which are receptors for NGF and BDNF, respectively, were targeted by JAK inhibitors. The binding of neurotrophins to their receptors activates several downstream pathways, including PI3K/Akt and MAPK, which are also important in the pathogenesis of AD, as previously described [57]. These findings demonstrate that JAK inhibitors may modulate both upstream and downstream signaling in the neurotrophin pathway to exert their effects in AD. Taken together, these findings suggest the potential therapeutic role of JAK inhibitors in AD through the regulation of several interconnected signaling pathways. By simultaneously targeting neuroinflammation, apoptosis, and synaptic dysfunction, these inhibitors may provide a multi-target therapeutic strategy, rather than acting on a single pathway.

Identifying key regulatory nodes is essential for understanding the effectiveness of drugs in the complex AD landscape. Among the target proteins identified in this study, AKT1, BCL2, SRC, STAT3, and TNF emerged as central hub proteins because of their high connectivity in the PPI network. These proteins participate in the neurotrophin, PI3K/Akt, and MAPK signaling pathways, which represent critical intersection points among neuroinflammation, apoptosis, and synaptic plasticity, highlighting their essential roles in mediating the anti-AD effects of these compounds. Specifically, STAT3 and TNF are recognized as the primary drivers of chronic neuroinflammatory responses and microglial activation in the brains of patients with AD [12,58]. AKT1 and BCL2 play pivotal roles in maintaining neuronal survival, regulating autophagy, and countering Aβ-induced cytotoxicity [59,60]. Furthermore, the SRC family of kinases is instrumental in mediating synaptic signaling and receptor trafficking, which are often impaired in neurodegenerative diseases [61]. By focusing on these five hub proteins, this study evaluated how Janus kinase (JAK) inhibitors might exert multi-target synergistic effects to stabilize the neuro-microenvironment and halt the progressive neurodegeneration characteristic of AD.

Transcriptomic validation showed that *AKT1*, *BCL2*, *SRC*, *STAT3*, and *TNF* were upregulated in the brains of patients with AD, particularly in the prefrontal cortex, compared with those in healthy controls. The consistent upregulation of all five hub genes in the prefrontal cortex, compared to the more variable patterns observed in the cerebellum and visual cortex, may reflect regional differences in AD-related molecular alterations. The prefrontal cortex is involved in higher-order cognitive functions, including memory and behavior, and undergoes substantial molecular and transcriptomic changes during AD progression [62,63]. Consistent with our findings, previous studies have reported that increased AKT1 activation in the prefrontal cortex is associated with faster cognitive decline in humans [64]. Similarly, the prefrontal cortex may be particularly susceptible to Aβ-related neurotoxicity, with increased TNF-α levels associated with impaired learning and memory in AD mice [65]. In contrast, the cerebellum and visual cortex are generally less vulnerable to classical AD pathology and are affected later in the disease progression [66]. Thus, the absence of significant changes in individual hub genes in these regions does not necessarily contradict their potential involvement in AD but may reflect region-specific differences in transcriptional regulation and disease-related responses. Notably, the allocortex, particularly the entorhinal cortex and hippocampus, is the most vulnerable brain region in AD [66]. However, appropriate public datasets for these brain regions with adequate sample sizes were unavailable for inclusion in the present study. Therefore, our findings should be interpreted primarily in the context of cortical AD pathology and may not fully capture region-specific molecular alterations in the brain of patients with AD. ROC curve analysis further demonstrated the ability of these hub genes to distinguish AD from control samples within the selected datasets, supporting their potential relevance to AD-associated gene expression patterns. In addition, the expression of these genes is regulated at both the transcriptional and post-transcriptional levels by multiple transcription factors and miRNAs. These regulatory mechanisms may affect the expression and activity of genes involved in neuroinflammation, neuronal survival, and synaptic plasticity, further contributing to the complex molecular signaling pathways underlying AD pathology.

Molecular docking analysis suggested favorable interactions between the five JAK inhibitor candidates and the five hub proteins, while NMA provided additional validation on the predicted flexibility and collective motions of the docked complexes. Among the JAK inhibitors, pacritinib exhibited favorable predicted binding affinities for both kinase proteins (AKT1, SRC, and STAT3) and non-kinase proteins (TNF and BCL2). However, these docking results do not demonstrate direct physical binding and require further in vitro and in vivo validation studies. Interestingly, previous studies have reported that pacritinib suppresses TNF and STAT3 expression in myelofibrosis and glioblastoma models, respectively [67,68]. Notably, the predicted interactions of JAK inhibitors, particularly pacritinib, with these five hub proteins may contribute to the modulation of neuroinflammatory and neurotrophin-related signaling pathways rather than indicating direct inhibition. Overall, these findings suggest potential mechanisms by which JAK inhibitors may influence AD-related pathways; however, further experimental studies are needed for confirmation.

ADMET prediction using the pkCSM platform showed that the five JAK inhibitors had favorable pharmacokinetic profiles. As these drugs have previously received FDA approval for oral administration in the treatment of various diseases, they exhibit good Caco-2 permeability and intestinal absorption. In this study, we focused on the ability of these drugs to cross the BBB and penetrate the CNS. Pacritinib was the only drug meeting the criteria for BBB+ compounds, with a BBB permeability index (log BB) > −1.0 and a CNS permeability index (log PS) > −2.0, which have been used as cutoff values for predicting BBB permeability in previous machine-learning studies [69,70,71,72]. Although the negative log BB and log PS values predicted for pacritinib suggest limited BBB permeability and CNS exposure under normal conditions, BBB integrity may be compromised in the chronic neuroinflammatory environment of AD, potentially increasing its permeability to the CNS [73]. Moreover, a previous study suggested that pacritinib crosses the BBB and accumulates in the brains of mice with glioblastoma [74]. Although this finding was observed in a different disease model and cannot be directly extrapolated to AD, it provides experimental evidence that pacritinib may reach brain tissue under pathological conditions. These findings support further investigation of the potential of pacritinib to achieve CNS exposure in AD.

All five analyzed JAK inhibitors showed predicted hepatotoxicity, and several were predicted to interact with major CYP enzymes, such as CYP1A2 and CYP3A4. Although these computational predictions do not establish clinically relevant hepatotoxicity or CYP inhibition, they raise important safety concerns regarding potential long-term use in AD, particularly in elderly patients with AD who may have age-related changes in hepatic function and experience polypharmacy. Therefore, the potential CNS benefits of JAK inhibitors, particularly pacritinib, should be evaluated along with their predicted safety liabilities. Further pharmacokinetic, toxicological, and drug–drug interaction studies are needed to determine whether pacritinib can achieve adequate CNS exposure while maintaining an acceptable safety profile in humans.

The current study had some limitations. Transcriptomic analysis using the public GEO database may be biased because only datasets with large sample sizes (*n* > 100 per group) were included. Several datasets from primary AD-affected regions, particularly the hippocampus and entorhinal cortex, were excluded because of limited sample sizes (*n* < 20 per group), which could reduce the statistical power and robustness of the differential expression analysis. Although our study included data from the prefrontal cortex (GSE33000), an important brain region affected by AD, the findings may not fully capture region-specific inflammatory alterations in other key AD-affected regions, highlighting the need for further validation. Another limitation is that relevant covariates, including age, sex, post-mortem interval, neuropathological stage, and technical batch, were not adjusted for in the present analysis, which may have influenced the observed gene expression profiles. Moreover, GSE44768 and GSE44771 represent different brain regions obtained from the same donor cohort and therefore do not constitute fully independent validation cohorts. In this study, we used a SwissTargetPrediction probability threshold of > 0.1. Although this is consistent with previous computational studies, this cutoff may include lower-confidence predicted targets. As a sensitivity analysis, applying a stricter threshold (>0.5) substantially reduced the number of predicted targets and altered the resulting PPI network and hub protein rankings, indicating that hub-target identification is partly dependent on the selected prediction threshold and should be interpreted cautiously. Therefore, these findings should be considered as hypothesis-generating evidence and require experimental validation. Molecular docking, NMA simulations, and ADMET predictions, while indicating potential drug–target interactions and pharmacokinetic properties, are computational methods that may not fully capture the complex physiological environment of cells and organisms. Therefore, in vitro and in vivo disease models are necessary to confirm these results and provide strong evidence for the potential use of JAK inhibitors in AD treatment. Unexpectedly, a recent study indicated that prolonged use of pan-JAK inhibitors, such as tofacitinib, is linked to memory issues in patients with chronic inflammatory conditions, particularly rheumatoid arthritis [75]. This suggests that JAK inhibitors should be adjusted within a precise therapeutic window to optimize their anti-neuroinflammatory effects while preserving normal memory function.

## 5. Conclusions

This study is the first to systematically identify the primary targets and signaling pathways that may mediate the potential effects of JAK inhibitors in AD treatment using an integrated network pharmacology, molecular docking analysis, and transcriptomic data analysis. Our results showed that JAK inhibitors target multiple genes and proteins involved in the neurotrophin, PI3K/Akt, and MAPK signaling pathways, which are closely associated with AD pathogenesis. PPI network analysis identified AKT1, BCL2, SRC, STAT3, and TNF as highly connected targets with broad roles in multiple cellular processes. Additionally, molecular docking and NMA simulations predicted favorable interactions between the JAK inhibitors and core proteins, while NMA provided insights into the structural flexibility of the docked complexes. Among the selected JAK inhibitors, pacritinib showed relatively stronger predicted interactions with the target proteins and favorable predicted pharmacokinetic properties, supporting its potential for further investigation in AD treatment. Further experimental studies are required to validate these computational findings and clarify the therapeutic potential of JAK inhibitors in AD.

## Figures and Tables

**Table 1:** Information on JAK inhibitors used for screening.

Drug	Specific Target	DrugBank ID	PubChem CID	FDA-Approved Treatment
Abrocitinib	JAK1	DB14973	78323835	refractory, moderate-to-severe atopic dermatitis in adults
Baricitinib	JAK1/JAK2	DB11817	44205240	rheumatoid arthritis in adults
Deucravacitinib	TYK2	DB16650	134821691	moderate-to-severe plaque psoriasis
Deuruxolitinib	JAK1/JAK2	DB18847	72704611	severe alopecia areata
Fedratinib	JAK2	DB12500	16722836	intermediate-2 and high-risk primary and secondary myelofibrosis
Momelotinib	JAK1/JAK2	DB11763	25062766	intermediate or high-risk myelofibrosis
Pacritinib	JAK2	DB11697	46216796	primary and secondary myelofibrosis in adults
Ritlecitinib	JAK3	DB14924	118115473	severe alopecia areata in adults and adolescents 12 years and older
Ruxolitinib	JAK1/JAK2	DB08877	25126798	myelofibrosis and polycythemia vera in adults, steroid-refractory acute graft-versus-host disease in adults and children
Tofacitinib	JAK1/JAK3	DB08895	9926791	moderate to severe rheumatoid arthritis
Upadacitinib	JAK1	DB15091	58557659	active rheumatoid arthritis, psoriatic arthritis, atopic dermatitis, ulcerative colitis, and ankylosing spondylitis

**Table 2:** Clinical characteristics of the control and AD groups in the selected microarray datasets.

Characteristics		GSE33000	GSE44768	GSE44771
Group	Normal control	157 (33.62%)	101 (43.91%)	101 (43.91%)
	AD	310 (66.38%)	129 (56.09%)	129 (56.09%)
Age	Mean ± SD	74.86 ± 12.32	72.23 ± 13.41	72.23 ± 13.41
Gender	Female	209 (44.75%)	86 (37.39%)	86 (37.39%)
	Male	258 (55.25%)	144 (62.61%)	144 (62.61%)
Brain region		Prefrontal cortex	Cerebellum	Visual cortex

**Table 3:** Grid box settings for molecular docking analysis.

Protein	PDB ID	Resolution (Å)	Center x (Å)	Center y (Å)	Center z (Å)	Size x (Å)	Size y (Å)	Size z (Å)
AKT1	4EKL	2.00	27.198	4.027	12.025	23	19	22
BCL2	6GL8	1.40	17.189	2.647	15.647	21	18	20
SRC	8JF3	2.85	−1.272	33.617	13.127	20	20	20
STAT3	6NJS	2.70	13.498	54.118	0.101	20	20	20
TNF	2AZ5	2.10	−19.162	74.451	33.837	17	16	16

**Table 4:** Topological analysis of the drugs in the drug–target network.

No.	Drug	Degree	Betweenness Centrality	Closeness Centrality
1	Baricitinib	50	0.226684919	0.420924574
2	Momelotinib	49	0.295371044	0.420924574
3	Fedratinib	48	0.221096997	0.418886199
4	Pacritinib	40	0.23089212	0.403263403
5	Upadacitinib	32	0.16509733	0.388764045
6	Deucravacitinib	29	0.153035391	0.383592018
7	Deuruxolitinib	28	0.046159284	0.381898455
8	Ruxolitinib	28	0.046159284	0.381898455
9	Ritlecitinib	23	0.07678396	0.373650108
10	Tofacitinib	22	0.073389897	0.372043011
11	Abrocitinib	8	0.009757788	0.350912779

**Table 5:** Topological analysis of the 10 hub proteins in the PPI network.

No.	Protein	Degree	Betweenness Centrality	Closeness Centrality
1	AKT1	104	0.063618066	0.723214286
2	SRC	103	0.074595117	0.726457399
3	TNF	102	0.085024906	0.723214286
4	BCL2	98	0.037831146	0.713656388
5	STAT3	96	0.036270176	0.704347826
6	CASP3	91	0.029533716	0.692307692
7	EGFR	89	0.027108592	0.686440678
8	ESR1	81	0.026694189	0.655870445
9	MTOR	79	0.017530369	0.655870445
10	CCND1	73	0.017085337	0.637795276

**Table 6:** Interaction profiles of representative protein–compound complexes.

Protein	Compound	Interaction Type	Residue	Distance (Å)
AKT1	Pacritinib	Conventional hydrogen bond	PHE161	2.24
		Carbon hydrogen bond	ASP292	3.49
		Carbon hydrogen bond	GLU191	3.6
		Carbon hydrogen bond	GLU278	3.53
		Pi donor hydrogen bond	PHE161	2.96
		Pi-Alkyl	PHE161	4.97
		Pi-Anion	ASP292	3.58
		Pi-Cation	LYS179	4.75
		Pi-Pi T shaped	PHE161	4.76
BCL2	Momelotinib	Conventional hydrogen bond	GLN118	2.04
		Conventional hydrogen bond	ARG146	2.34
		Carbon hydrogen bond	ASP111	3.45
		Carbon hydrogen bond	ASP111	3.50
		Pi donor hydrogen bond	GLN118	3.02
SRC	Pacritinib	Carbon hydrogen bond	SER525	3.24
		Carbon hydrogen bond	THR526	3.38
		Alkyl	LYS354	5.45
		Alkyl	PRO528	4.66
		Pi-Alkyl	PRO528	5.39
STAT3	Momelotinib	Conventional hydrogen bond	PRO639	2.22
		Carbon hydrogen bond	TYR640	3.79
		Carbon hydrogen bond	SER614	3.63
		Akyl	ILE653	5.31
TNF	Pacritinib	Akyl	LEU57 (chain B)	5.50
		Pi-Akyl	LEU57 (chain A)	5.47

**Table 7:** ADMET prediction of potential candidates for AD.

Parameter		Baricitinib	Fedratinib	Momelotinib	Pacritinib	Upadacitinib
**Absorption**	Caco2 permeability (log P_app_)	0.349	0.5	1.008	1.356	1.271
	Intestinal absorption (%)	79.6	83.08	86.835	94.068	88.216
**Distribution**	BBB permeability (log BB)	−1.346	−1.344	−0.43	−0.267	−1.245
	CNS permeability (log PS)	−3.606	−2.574	−2.395	−1.924	−3.168
**Metabolism**	CYP1A2 inhibitor	No	No	Yes	No	Yes
	CYP2C19 inhibitor	No	Yes	Yes	No	No
	CYP2C9 inhibitor	No	Yes	No	Yes	No
	CYP2D6 inhibitor	No	No	No	No	No
	CYP3A4 inhibitor	No	Yes	Yes	Yes	No
**Excretion**	Total clearance (log mL/min/kg)	0.875	0.363	0.344	0.671	0.357
	Renal OCT2 substrate	No	No	No	No	No
**Toxicity**	Ames toxicity (mutagenicity)	No	No	Yes	No	No
	Hepatotoxicity	Yes	Yes	Yes	Yes	Yes

P_app_, apparent permeability coefficient; BBB, blood–brain barrier; BB, brain–to–blood concentration ratio; CNS, central nervous system; PS, permeability–surface area product; CYP, cytochrome P450; OCT2, organic cation transporter 2.

## Data Availability

Data are contained within the article or Supplementary Materials.

## Supplementary Materials

The following supporting information can be downloaded at https://www.mdpi.com/article/10.3390/medicina62091674/s1, Figure S1: Boxplots showing the distribution of normalized sample data across the three datasets using GEO2R. (A) GSE33000. (B) GSE44768. (C) GSE44771. Green indicates the AD group, and purple indicates the control group. Figure S2: Expression of JAK members in different brain regions in controls and patients with AD. The levels of JAK1, JAK2, JAK3, and TYK2 were retrieved and analyzed from the GEO datasets: (A) GSE33000 (prefrontal cortex), (B) GSE44768 (cerebellum), and (C) GSE44771 (visual cortex). Statistical analysis was conducted using multiple unpaired *t*-tests with Benjamini-Krieger-Yekutieli FDR correction (q-value); Figure S3: Neurotrophin signaling pathway. The targets of JAK inhibitors are outlined with bold black borders; Figure S4: PPI network of AD-related targets using a SwissTargetPrediction probability threshold of >0.5. (A) PPI network. (B) Subnetwork of the top five hub proteins; Figure S5: Redocking results of the five target proteins and the native ligands. (A) AKT1. (B) BCL2. (C) SRC. (D) STAT3. (E) TNF. The green indicates original ligands and the blue indicates redocked ligands. Figure S6: Molecular mobility of docked complexes. (A) AKT1–Pacritinib. (B) BCL2–Momelotinib. (C) SRC–Pacritinib. (D) STAT3–Momelotinib. (E) TNF–Pacritinib. The arrows indicate the directions of motion; Figure S7: NMA simulation of the docked complexes. Deformation and B-factor plots for (A) AKT1–Pacritinib, (B) BCL2–Momelotinib, (C) SRC–Pacritinib, (D) STAT3–Momelotinib, and (E) TNF–Pacritinib; Table S1: Predicted targets of JAK inhibitors obtained from SwissTargetPrediction; Table S2: AD-related targets retrieved from GeneCards database; Table S3: Predicted targets of JAK inhibitors obtained from SwissTargetPrediction using a probability threshold of >0.5; Table S4: GO biological process terms for potential targets of JAK inhibitors; Table S5: GO cellular component terms for potential targets of JAK inhibitors; Table S6: GO molecular function terms for potential targets of JAK inhibitors; Table S7: KEGG pathways for potential targets of JAK inhibitors; Table S8: Topological analysis of PPI network using different cytoHubba algorithms; Table S9: Docking validation results of native ligands against target proteins based on docking scores and RMSD values; Table S10: Eigenvalues for protein–ligand complexes predicted by NMA using iMODS.

## Abbreviations

The following abbreviations are used in this manuscript:

AD	Alzheimer’s disease
Aβ	Amyloid beta
NFT	Neurofibrillary tangles
JAK	Janus kinase
STAT	Signal transducer and activator of transcription
TYK2	Tyrosine kinase 2
KEGG	Kyoto Encyclopedia of Genes and Genomes
GO	Gene ontology
BP	Biological process
CC	Cellular component
MF	Molecular function
PI3K/Akt	Phosphatidylinositol 3-kinase/protein kinase B
MAPK	Mitogen-activated protein kinase
PPI	Protein–protein interaction
TNF	Tumor necrosis factor
ROC	Receiver operating characteristic
NMA	Normal mode analysis
ADMET	Absorption, distribution, metabolism, excretion, and toxicity

## References

[1] Scheltens P., De Strooper B., Kivipelto M., Holstege H., Chételat G., Teunissen C.E., Cummings J., Van der Flier W.M. (2021). Alzheimer’s disease. Lancet.

[2] Sadigh-Eteghad S., Sabermarouf B., Majdi A., Talebi M., Farhoudi M., Mahmoudi J. (2015). Amyloid-beta: A crucial factor in Alzheimer’s disease. Med. Princ. Pract..

[3] Heneka M.T., Carson M.J., El Khoury J., Landreth G.E., Brosseron F., Feinstein D.L., Jacobs A.H., Wyss-Coray T., Vitorica J., Ransohoff R.M. (2015). Neuroinflammation in Alzheimer’s disease. Lancet Neurol..

[4] Parsons C.G., Danysz W., Dekundy A., Pulte I. (2013). Memantine and cholinesterase inhibitors: Complementary mechanisms in the treatment of Alzheimer’s disease. Neurotox. Res..

[5] Abouelmagd M.E., Almosilhy N.A., Makhlouf H.A., Hassan A.K., Osman A.S.A., Hindawi M.D., Elshahat A., Alnajjar A.Z., Mustafa M.M.M., Abdelsalam O.K. (2025). Comparative safety of cholinesterase inhibitors and memantine for dementia: A protocol for a network meta-analysis of randomized controlled trials. Syst. Rev..

[6] van Dyck C.H., Swanson C.J., Aisen P., Bateman R.J., Chen C., Gee M., Kanekiyo M., Li D., Reyderman L., Cohen S. (2023). Lecanemab in Early Alzheimer’s Disease. N. Engl. J. Med..

[7] Sims J.R., Zimmer J.A., Evans C.D., Lu M., Ardayfio P., Sparks J., Wessels A.M., Shcherbinin S., Wang H., Monkul Nery E.S. (2023). Donanemab in Early Symptomatic Alzheimer Disease: The TRAILBLAZER-ALZ 2 Randomized Clinical Trial. JAMA.

[8] Thakur S., Dhapola R., Sarma P., Medhi B., Reddy D.H. (2023). Neuroinflammation in Alzheimer’s disease: Current progress in molecular signaling and therapeutics. Inflammation.

[9] Abdullah S.K., See-Too W.S., Mohd Mohidin T.B., Mohan G. (2026). Neuroinflammation as a central mechanism in Alzheimer’s disease: Therapeutic insights from Schiff base derivatives. Molecules.

[10] Rusek M., Smith J., El-Khatib K., Aikins K., Czuczwar S.J., Pluta R. (2023). The role of the JAK/STAT signaling pathway in the pathogenesis of Alzheimer’s disease: New potential treatment target. Int. J. Mol. Sci..

[11] Yan Z., Gibson S.A., Buckley J.A., Qin H., Benveniste E.N. (2018). Role of the JAK/STAT signaling pathway in regulation of innate immunity in neuroinflammatory diseases. Clin. Immunol..

[12] Al-Kuraishy H.M., Sulaiman G.M., Mohammed H.A., Saad H.M., Al-Gareeb A.I., Albuhadily A.K. (2025). Targeting the JAK/STAT3/SOCS signaling pathway in Alzheimer’s disease. Inflammopharmacology.

[13] Jain M., Singh M.K., Shyam H., Mishra A., Kumar S., Kumar A., Kushwaha J. (2021). Role of JAK/STAT in the neuroinflammation and its association with neurological disorders. Ann. Neurosci..

[14] Nevado-Holgado A.J., Ribe E., Thei L., Furlong L., Mayer M.-A., Quan J., Richardson J.C., Cavanagh J., Consortium N., Lovestone S. (2019). Genetic and real-world clinical data, combined with empirical validation, nominate Jak-Stat signaling as a target for Alzheimer’s disease therapeutic development. Cells.

[15] Zhang B., Gaiteri C., Bodea L.G., Wang Z., McElwee J., Podtelezhnikov A.A., Zhang C., Xie T., Tran L., Dobrin R. (2013). Integrated systems approach identifies genetic nodes and networks in late-onset Alzheimer’s disease. Cell.

[16] Ben Haim L., Ceyzériat K., Carrillo-de Sauvage M.A., Aubry F., Auregan G., Guillermier M., Ruiz M., Petit F., Houitte D., Faivre E. (2015). The JAK/STAT3 pathway is a common inducer of astrocyte reactivity in Alzheimer’s and Huntington’s diseases. J. Neurosci..

[17] Pushpakom S., Iorio F., Eyers P.A., Escott K.J., Hopper S., Wells A., Doig A., Guilliams T., Latimer J., McNamee C. (2019). Drug repurposing: Progress, challenges and recommendations. Nat. Rev. Drug Discov..

[18] Shawky A.M., Almalki F.A., Abdalla A.N., Abdelazeem A.H., Gouda A.M. (2022). A comprehensive overview of globally approved JAK inhibitors. Pharmaceutics.

[19] Chikhoune L., Poggi C., Moreau J., Dubucquoi S., Hachulla E., Collet A., Launay D. (2025). JAK inhibitors (JAKi): Mechanisms of action and perspectives in systemic and autoimmune diseases. Rev. Med. Interne.

[20] Knox C., Wilson M., Klinger C.M., Franklin M., Oler E., Wilson A., Pon A., Cox J., Chin N.E., Strawbridge S.A. (2024). DrugBank 6.0: The DrugBank knowledgebase for 2024. Nucleic Acids Res..

[21] Daina A., Michielin O., Zoete V. (2019). SwissTargetPrediction: Updated data and new features for efficient prediction of protein targets of small molecules. Nucleic Acids Res..

[22] Stelzer G., Rosen N., Plaschkes I., Zimmerman S., Twik M., Fishilevich S., Stein T.I., Nudel R., Lieder I., Mazor Y. (2016). The GeneCards suite: From gene data mining to disease genome sequence analyses. Curr. Protoc. Bioinform..

[23] Liu X., Ren Y., Qin S., Yang Z. (2024). Exploring the mechanism of 6-Methoxydihydrosanguinarine in the treatment of lung adenocarcinoma based on network pharmacology, molecular docking and experimental investigation. BMC Complement. Med. Ther..

[24] Que W., Chen M., Yang L., Zhang B., Zhao Z., Liu M., Cheng Y., Qiu H. (2021). A network pharmacology-based investigation on the bioactive ingredients and molecular mechanisms of Gelsemium elegans Benth against colorectal cancer. BMC Complement. Med. Ther..

[25] Zhang C., Zhang J. (2026). 6PPD-quinone exposure and Alzheimer’s disease: Insights from integrative network pharmacology, transcriptomics, machine learning, and molecular docking. Open Med..

[26] Yang H., Cao J., Zhou L., Chen J., Tang J., Chen J., Yin L., Xie L., Li J., Luo J. (2025). Exploring the Cardioprotective Mechanisms of Ligusticum wallichii in Myocardial Infarction Through Network Pharmacology and Experimental Validation. Drug Des. Devel Ther..

[27] Shannon P., Markiel A., Ozier O., Baliga N.S., Wang J.T., Ramage D., Amin N., Schwikowski B., Ideker T. (2003). Cytoscape: A software environment for integrated models of biomolecular interaction networks. Genome Res..

[28] Huang D.W., Sherman B.T., Tan Q., Collins J.R., Alvord W.G., Roayaei J., Stephens R., Baseler M.W., Lane H.C., Lempicki R.A. (2007). The DAVID gene functional classification tool: A novel biological module-centric algorithm to functionally analyze large gene lists. Genome Biol..

[29] Tang D., Chen M., Huang X., Zhang G., Zeng L., Zhang G., Wu S., Wang Y. (2023). SRplot: A free online platform for data visualization and graphing. PLoS ONE.

[30] Mehryary F., Nastou K., Ohta T., Jensen L.J., Pyysalo S. (2024). STRING-ing together protein complexes: Corpus and methods for extracting physical protein interactions from the biomedical literature. Bioinformatics.

[31] Chin C.-H., Chen S.-H., Wu H.-H., Ho C.-W., Ko M.-T., Lin C.-Y. (2014). cytoHubba: Identifying hub objects and sub-networks from complex interactome. BMC Syst. Biol..

[32] Barrett T., Edgar R., Bina M. (2006). Mining microarray data at NCBI’s Gene Expression Omnibus (GEO). Gene Mapping, Discovery, and Expression: Methods and Protocols.

[33] Zhang Q., Liu W., Zhang H.-M., Xie G.-Y., Miao Y.-R., Xia M., Guo A.-Y. (2020). hTFtarget: A comprehensive database for regulations of human transcription factors and their targets. Genom. Proteom. Bioinform..

[34] Ritchie W., Flamant S., Rasko J.E. (2009). Predicting microRNA targets and functions: Traps for the unwary. Nat. Methods.

[35] Trott O., Olson A.J. (2010). AutoDock Vina: Improving the speed and accuracy of docking with a new scoring function, efficient optimization, and multithreading. J. Comput. Chem..

[36] Kim S., Chen J., Cheng T., Gindulyte A., He J., He S., Li Q., Shoemaker B.A., Thiessen P.A., Yu B. (2023). PubChem 2023 update. Nucleic Acids Res..

[37] Rose Y., Duarte J.M., Lowe R., Segura J., Bi C., Bhikadiya C., Chen L., Rose A.S., Bittrich S., Burley S.K. (2021). RCSB Protein Data Bank: Architectural advances towards integrated searching and efficient access to macromolecular structure data from the PDB archive. J. Mol. Biol..

[38] Yuan S., Chan H.S., Hu Z. (2017). Using PyMOL as a platform for computational drug design. Wiley Interdiscip. Rev. Comput. Mol. Sci..

[39] Baroroh U., Biotek M., Muscifa Z.S., Destiarani W., Rohmatullah F.G., Yusuf M. (2023). Molecular interaction analysis and visualization of protein-ligand docking using Biovia Discovery Studio Visualizer. Indones. J. Comput. Biol..

[40] López-Blanco J.R., Aliaga J.I., Quintana-Ortí E.S., Chacón P. (2014). iMODS: Internal coordinates normal mode analysis server. Nucleic Acids Res..

[41] Pires D.E.V., Blundell T.L., Ascher D.B. (2015). pkCSM: Predicting small-molecule pharmacokinetic and toxicity properties using graph-based signatures. J. Med. Chem..

[42] Zhou S., Wang F., Hsieh T.C., Wu J.M., Wu E. (2013). Thalidomide-a notorious sedative to a wonder anticancer drug. Curr. Med. Chem..

[43] Hennekens C.H., Dyken M.L., Fuster V. (1997). Aspirin as a therapeutic agent in cardiovascular disease. Circulation.

[44] Yang L., Liu Y., Wang Y., Li J., Liu N. (2021). Azeliragon ameliorates Alzheimer’s disease via the Janus tyrosine kinase and signal transducer and activator of transcription signaling pathway. Clinics.

[45] Sui S., Lv H. (2024). Cognitive improving actions of tofacitinib in a mouse model of Alzheimer disease involving TNF-α, IL-6, PI3K-Akt and GSK-3β signalling pathway. Int. J. Neurosci..

[46] Hindam M.O., Ahmed L.A., El Sayed N.S., Khattab M., Sallam N.A. (2024). Repositioning of baricitinib for management of memory impairment in ovariectomized/D-galactose treated rats: A potential role of JAK2/STAT3-PI3K/AKT/mTOR signaling pathway. Life Sci..

[47] Richardson P.J., Smith D.P., de Giorgio A., Snetkov X., Almond-Thynne J., Cronin S., Mead R.J., McDermott C.J., Shaw P.J. (2023). Janus kinase inhibitors are potential therapeutics for amyotrophic lateral sclerosis. Transl. Neurodegener..

[48] Bhadauriya M.S., Singh H., Suri M., Hanifa M., Bali A. (2025). JAK/STAT inhibitors mitigate sepsis-associated cerebral and cognitive injury. Fundam. Clin. Pharmacol..

[49] Almasoudi S.H., Al-kuraishy H.M., Al-Gareeb A.I., Eliwa D., Alexiou A., Papadakis M., Batiha G.E.-S. (2025). Role of mitogen-activated protein kinase inhibitors in Alzheimer’s disease: Rouge of brain kinases. Brain Res. Bull..

[50] Alam J., Blackburn K., Patrick D. (2017). Neflamapimod: Clinical Phase 2b-Ready Oral Small Molecule Inhibitor of p38α to Reverse Synaptic Dysfunction in Early Alzheimer’s Disease. J. Prev. Alzheimers Dis..

[51] Min J., Zheng H., Xia H., Tian X., Liang M., Zhang J., Huang W. (2024). Ruxolitinib attenuates microglial inflammatory response by inhibiting NF-κB/MAPK signaling pathway. Eur. J. Pharmacol..

[52] Razani E., Pourbagheri-Sigaroodi A., Safaroghli-Azar A., Zoghi A., Shanaki-Bavarsad M., Bashash D. (2021). The PI3K/Akt signaling axis in Alzheimer’s disease: A valuable target to stimulate or suppress?. Cell Stress Chaperones.

[53] Brunet A., Datta S.R., Greenberg M.E. (2001). Transcription-dependent and -independent control of neuronal survival by the PI3K–Akt signaling pathway. Curr. Opin. Neurobiol..

[54] Ali T., Kim M.O. (2015). Melatonin ameliorates amyloid beta-induced memory deficits, tau hyperphosphorylation and neurodegeneration via PI 3/Akt/GS k3β pathway in the mouse hippocampus. J. Pineal Res..

[55] Cianciulli A., Porro C., Calvello R., Trotta T., Lofrumento D.D., Panaro M.A. (2020). Microglia Mediated Neuroinflammation: Focus on PI3K Modulation. Biomolecules.

[56] Allen S.J., Watson J.J., Dawbarn D. (2011). The neurotrophins and their role in Alzheimer’s disease. Curr. Neuropharmacol..

[57] Nguyen N., Lee S.B., Lee Y.S., Lee K.H., Ahn J.Y. (2009). Neuroprotection by NGF and BDNF against neurotoxin-exerted apoptotic death in neural stem cells are mediated through Trk receptors, activating PI3-kinase and MAPK pathways. Neurochem. Res..

[58] Zhang Z., Liu X., Zhang S., Song Z., Lu K., Yang W. (2024). A review and analysis of key biomarkers in Alzheimer’s disease. Front. Neurosci..

[59] Chen S.Y., Gao Y., Sun J.Y., Meng X.L., Yang D., Fan L.H., Xiang L., Wang P. (2020). Traditional Chinese medicine: Role in reducing beta-amyloid, apoptosis, autophagy, neuroinflammation, oxidative stress, and mitochondrial dysfunction of Alzheimer’s disease. Front. Pharmacol..

[60] Mohamed Asik R., Suganthy N., Aarifa M.A., Kumar A., Szigeti K., Mathe D., Gulyas B., Archunan G., Padmanabhan P. (2021). Alzheimer’s disease: A molecular view of beta-amyloid induced morbific events. Biomedicines.

[61] Portugal C.C., Almeida T.O., Socodato R., Relvas J.B. (2022). Src family kinases (SFKs): Critical regulators of microglial homeostatic functions and neurodegeneration in Parkinson’s and Alzheimer’s diseases. FEBS J..

[62] Hanganu-Opatz I.L., Klausberger T., Sigurdsson T., Nieder A., Jacob S.N., Bartos M., Sauer J.-F., Durstewitz D., Leibold C., Diester I. (2023). Resolving the prefrontal mechanisms of adaptive cognitive behaviors: A cross-species perspective. Neuron.

[63] Yang F., Diao X., Wang F., Wang Q., Sun J., Zhou Y., Xie J. (2020). Identification of key regulatory genes and pathways in prefrontal cortex of Alzheimer’s disease. Interdiscip. Sci. Comput. Life Sci..

[64] Tong H., Petyuk V.A., Sendtner M., Sood A., Bennett D.A., Capuano A.W., Arvanitakis Z. (2025). Alzheimer’s disease-related cortical proteins modify the association of brain insulin signaling with cognitive decline. J. Alzheimers Dis..

[65] Medeiros R., Prediger R.D., Passos G.F., Pandolfo P., Duarte F.S., Franco J.L., Dafre A.L., Di Giunta G., Figueiredo C.P., Takahashi R.N. (2007). Connecting TNF-α signaling pathways to iNOS expression in a mouse model of Alzheimer’s disease: Relevance for the behavioral and synaptic deficits induced by amyloid β protein. J. Neurosci..

[66] Serrano-Pozo A., Frosch M.P., Masliah E., Hyman B.T. (2011). Neuropathological alterations in Alzheimer disease. Cold Spring Harb. Perspect. Med..

[67] Cuenca-Zamora E.J., Martínez C., Morales M.L., Guijarro-Carrillo P.J., López-Poveda M.J., Alcolea-Guardiola C., Vidal-Garrido N., Lozano M.L., Gonzalez-Conejero R., Teruel-Montoya R. (2024). Pacritinib prevents inflammation-driven myelofibrosis-like phenotype in a miR-146a(-/-) murine model. Biomed. Pharmacother..

[68] Chuang H.Y., Su Y.K., Liu H.W., Chen C.H., Chiu S.C., Cho D.Y., Lin S.Z., Chen Y.S., Lin C.M. (2019). Preclinical Evidence of STAT3 Inhibitor Pacritinib Overcoming Temozolomide Resistance via Downregulating miR-21-Enriched Exosomes from M2 Glioblastoma-Associated Macrophages. J. Clin. Med..

[69] Meng F., Xi Y., Huang J., Ayers P.W. (2021). A curated diverse molecular database of blood-brain barrier permeability with chemical descriptors. Sci. Data.

[70] Carpenter T.S., Kirshner D.A., Lau E.Y., Wong S.E., Nilmeier J.P., Lightstone F.C. (2014). A method to predict blood-brain barrier permeability of drug-like compounds using molecular dynamics simulations. Biophys. J..

[71] Suenderhauf C., Hammann F., Huwyler J. (2012). Computational Prediction of Blood-Brain Barrier Permeability Using Decision Tree Induction. Molecules.

[72] Chushak Y., Sterner T.R., Clewell R.A. (2026). Predicting blood-brain barrier permeability of chemicals by machine learning modeling. NAM J..

[73] Nehra G., Bauer B., Hartz A.M.S. (2022). Blood-brain barrier leakage in Alzheimer’s disease: From discovery to clinical relevance. Pharmacol. Ther..

[74] Jensen K.V., Cseh O., Aman A., Weiss S., Luchman H.A. (2017). The JAK2/STAT3 inhibitor pacritinib effectively inhibits patient-derived GBM brain tumor initiating cells in vitro and when used in combination with temozolomide increases survival in an orthotopic xenograft model. PLoS ONE.

[75] Duboëlle M., Lercara A., Pers Y.M., Michel C., Lepelley M., Valnet-Rabier M.B., Faillie J.L., Bres V., Palassin P. (2026). JAK Inhibitors and Memory Impairment: Disproportionality Analyses in the WHO Global Pharmacovigilance Database VigiBase. Fundam. Clin. Pharmacol..

