# Supplementary material for: Computational Repurposing of Janus Kinase Inhibitors as Potential Therapeutic Candidates for Alzheimer’s Disease
Source: Medicina (Kaunas). 2026 Aug 31;62(9):1674. doi: 10.3390/medicina62091674 (PMC13609782; doi:10.3390/medicina62091674)
Supplement: Supplementary file 1 [file medicina-62-01674-s001.zip › Supplementary Figures.pdf]

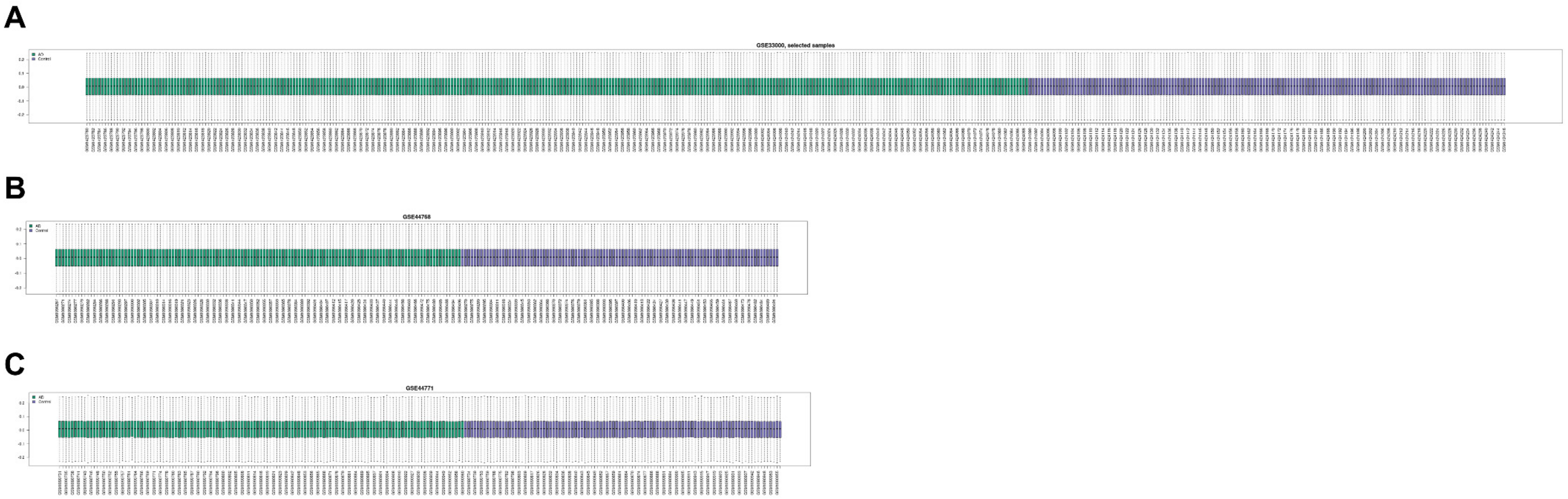

Figure S1. Boxplots showing the distribution of normalized sample data across the three datasets using GEO2R. (A) GSE33000. (B) GSE44768. (C) GSE44771. Green indicates the AD group and purple indicates the control group.

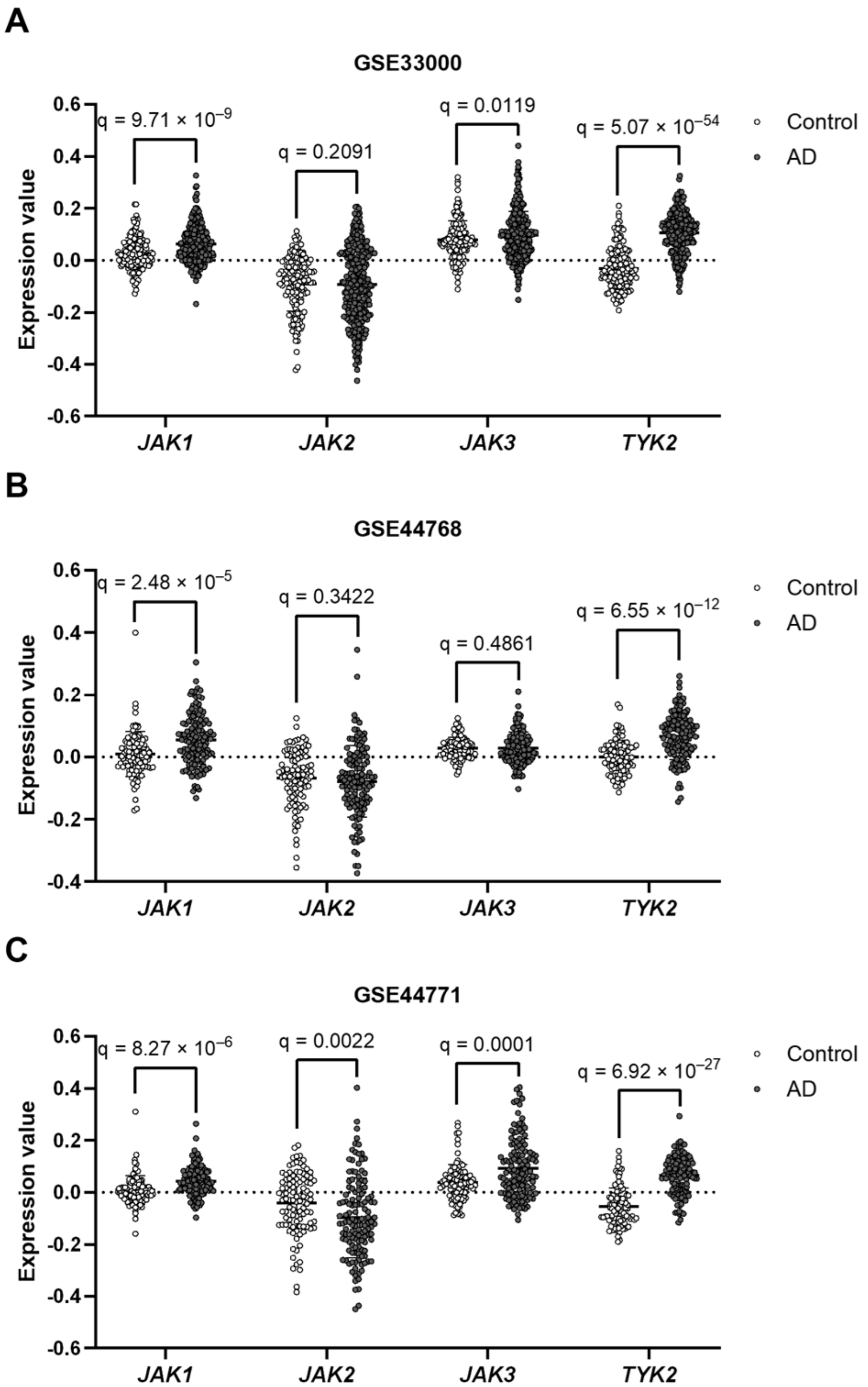

Figure S2. Expression of JAK members in different brain regions in controls and patients with AD. The levels of JAK1, JAK2, JAK3, and TYK2 were retrieved and analyzed from the GEO datasets: (A) GSE33000 (prefrontal cortex), (B) GSE44768 (cerebellum), and (C) GSE44771

(visual cortex). Statistical analysis was conducted using multiple unpaired t-test with Benjamini-Krieger-Yekutieli FDR correction (q-value).

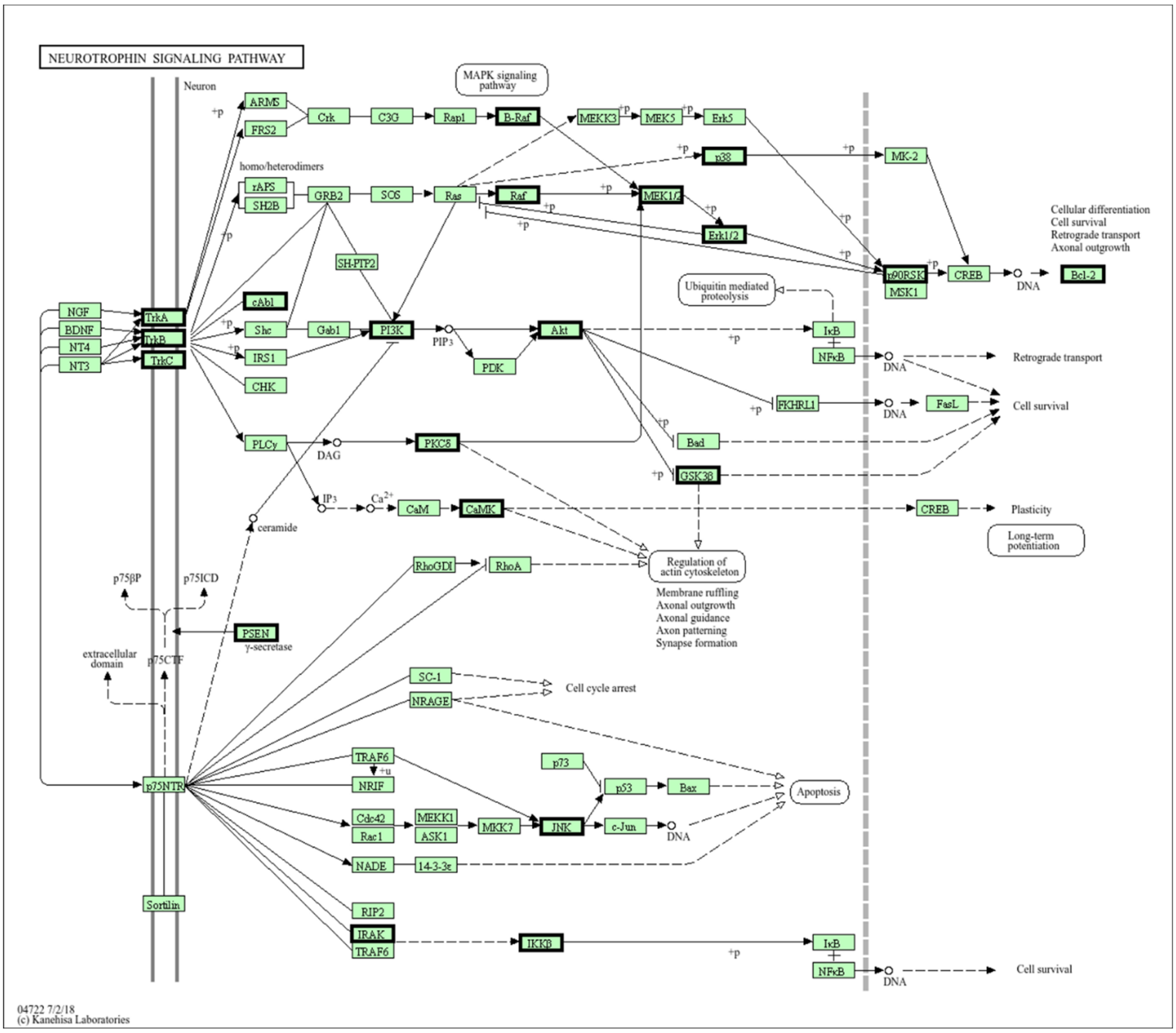

Figure S3. Neurotrophin signaling pathway. The targets of JAK inhibitors are outlined with bold black borders.

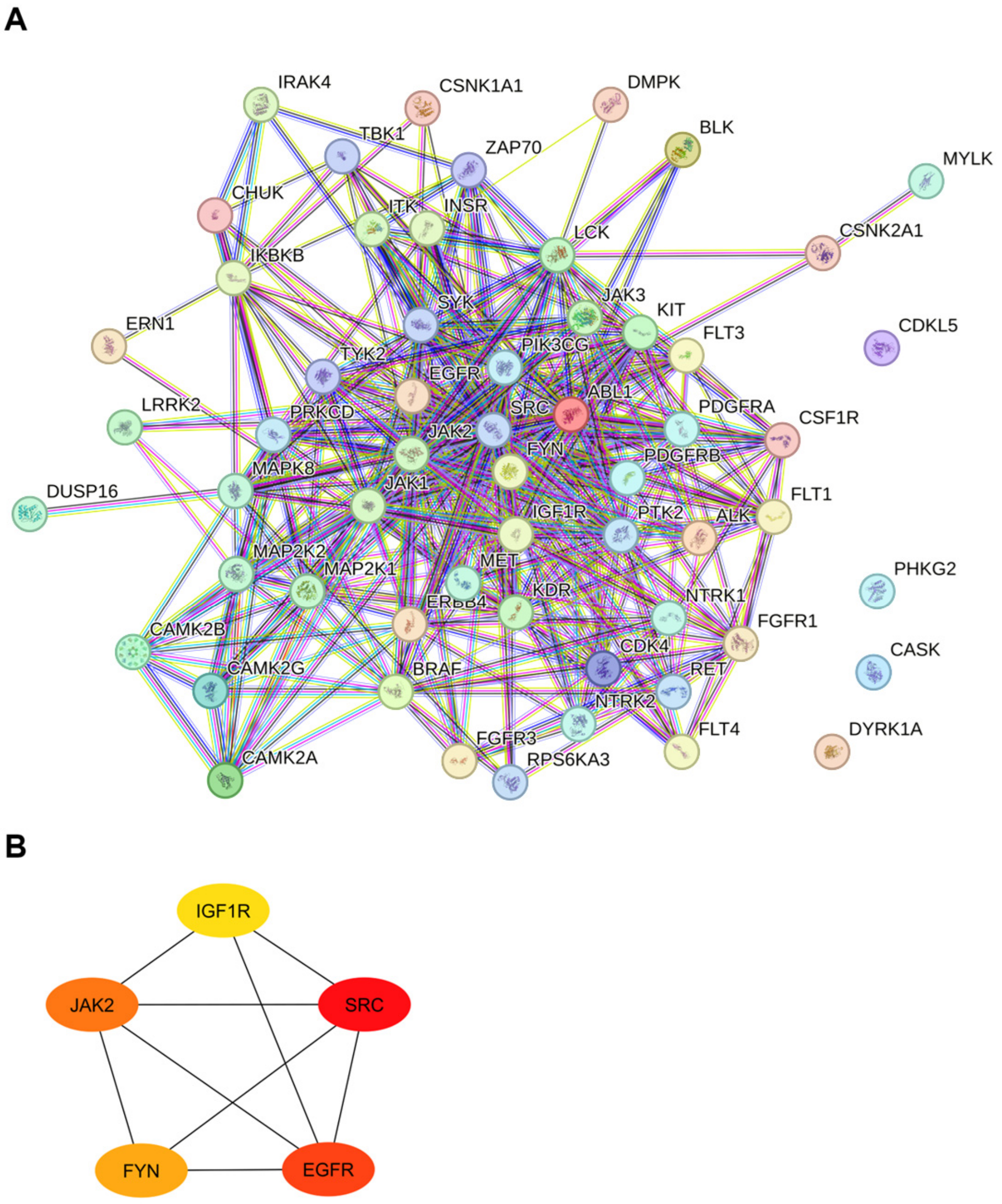

Figure S4. PPI network of AD-related targets using a SwissTargetPrediction probability threshold of >0.5. (A) PPI network. (B) Subnetwork of the top five hub proteins.

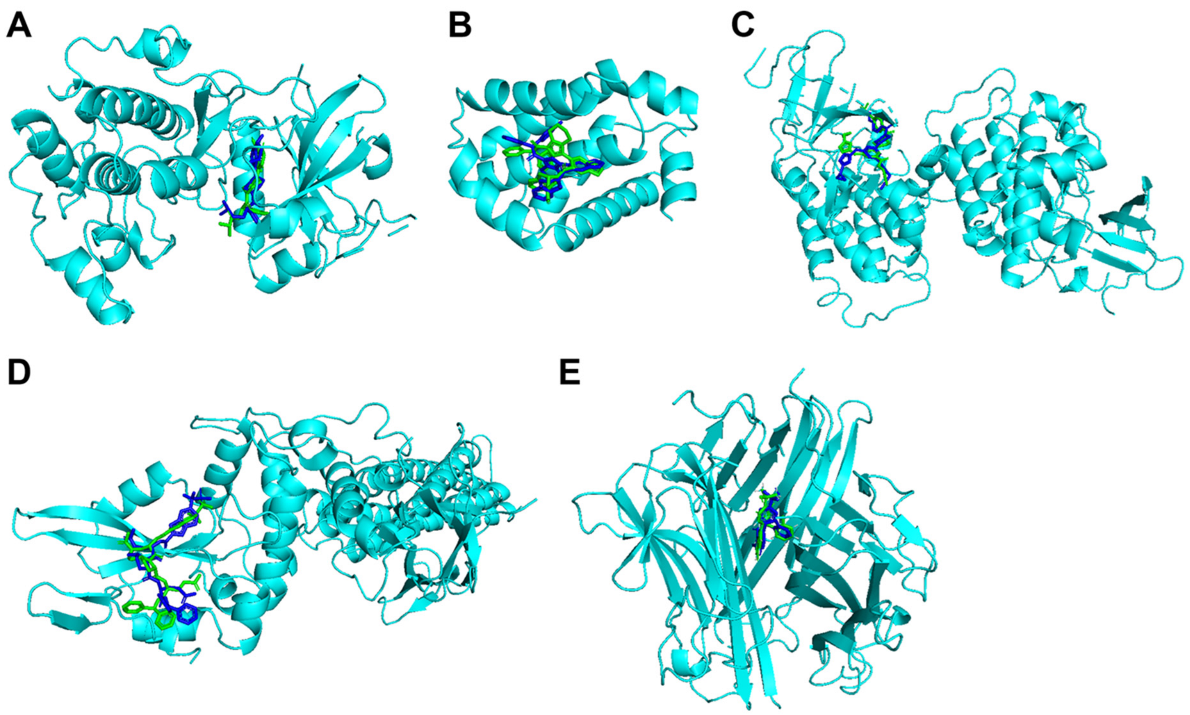

Figure S5. Redocking results of the five target proteins and the native ligands. (A) AKT1. (B) BCL2. (C) SRC. (D) STAT3. (E) TNF. The green indicates original ligands and the blue indicates redocked ligands.

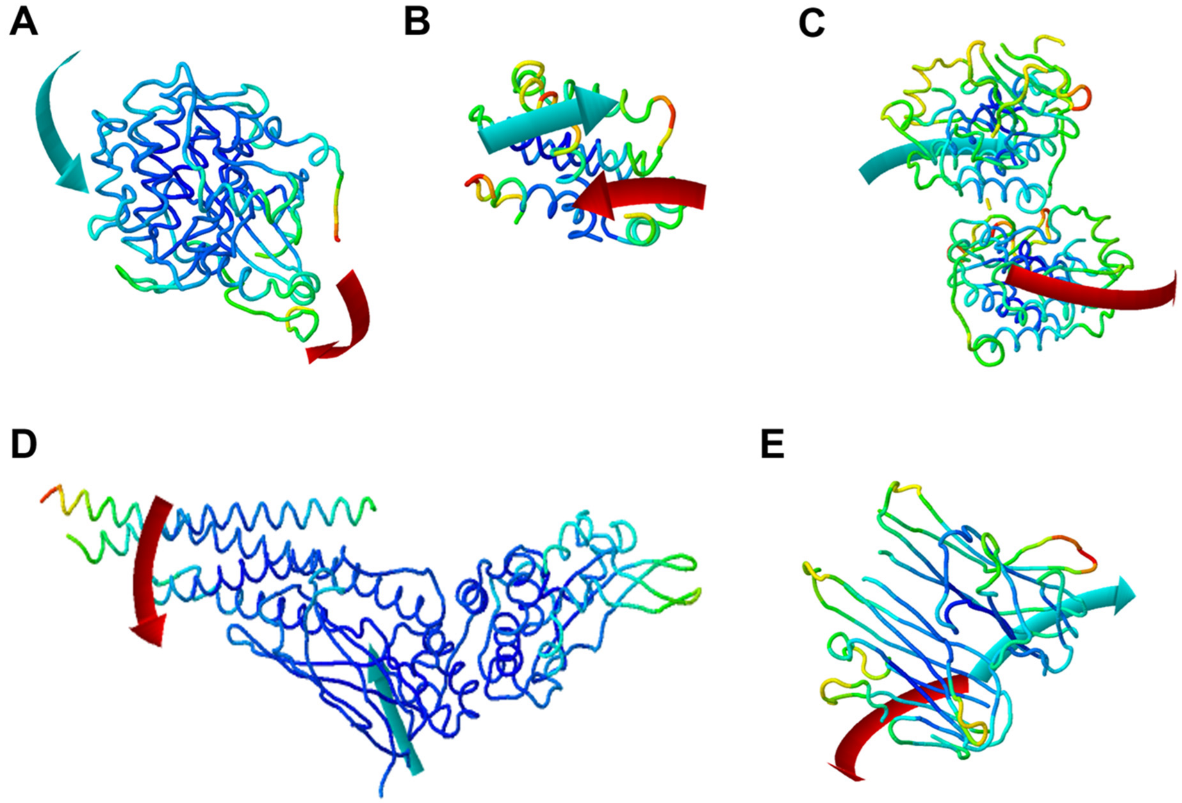

Figure S6. Molecular mobility of the docked complexes. (A) AKT1–Pacritinib. (B) BCL2–Momelotinib. (C) SRC–Pacritinib. (D) STAT3–Momelotinib. (E) TNF–Pacritinib. The arrows indicate the directions of motion.

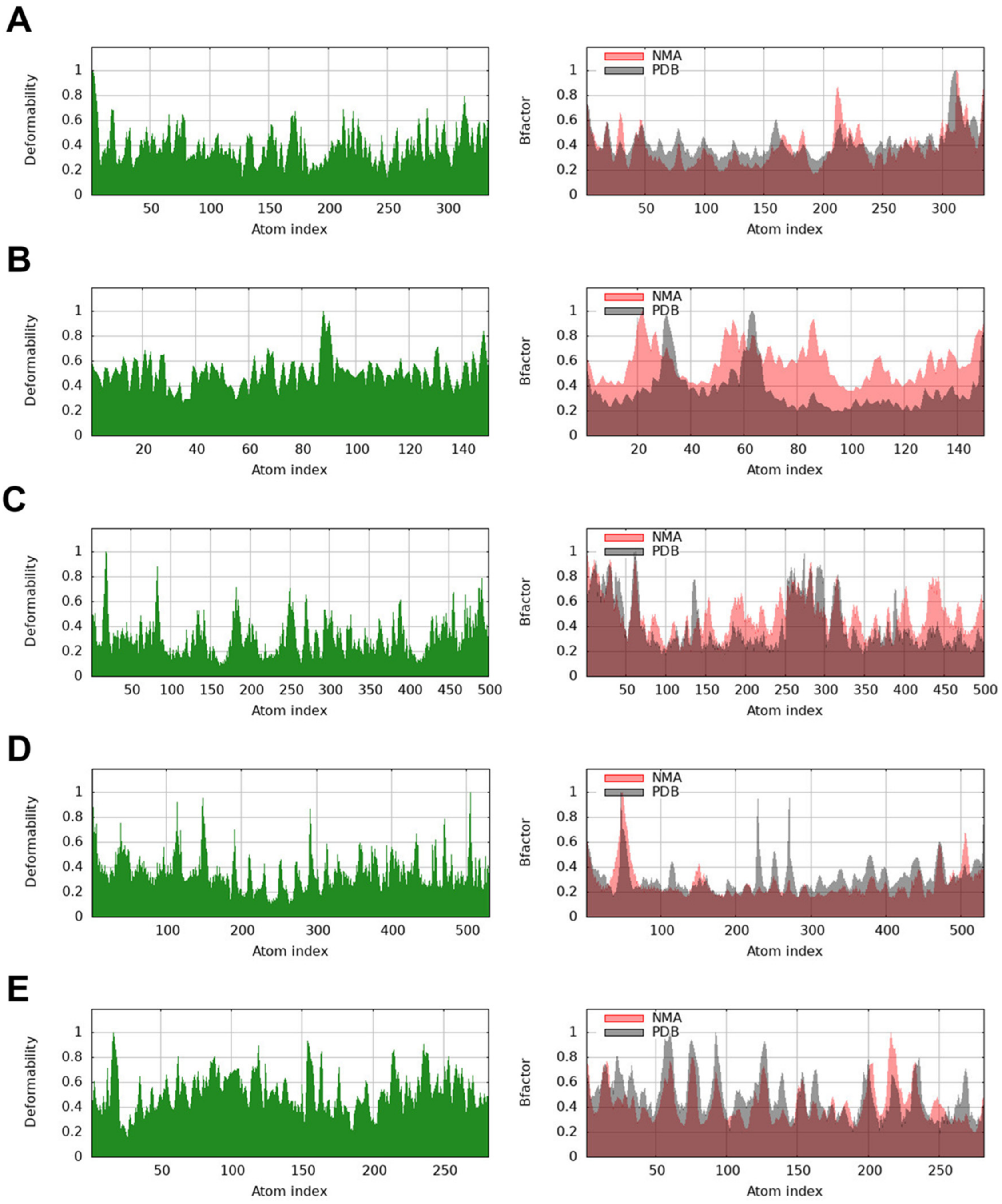

Figure S7. NMA simulation of the docked complexes. Deformation and B-factor plots for (A) AKT1–Pacritinib, (B) BCL2–Momelotinib, (C) SRC–Pacritinib, (D) STAT3–Momelotinib, and (E) TNF–Pacritinib.
